# sPGGM: a sample-perturbed Gaussian graphical model for identifying pre-disease stages and signaling molecules of disease progression

**DOI:** 10.1093/nsr/nwaf189

**Published:** 2025-05-14

**Authors:** Jiayuan Zhong, Junxian Li, Xuerong Gu, Dandan Ding, Fei Ling, Pei Chen, Rui Liu

**Affiliations:** School of Mathematics, Foshan University, Foshan 528000, China; School of Mathematics, South China University of Technology, Guangzhou 510640, China; School of Biology and Biological Engineering, South China University of Technology, Guangzhou 510640, China; Department of Oncology, First People's Hospital of Foshan, Foshan 528000, China; School of Biology and Biological Engineering, South China University of Technology, Guangzhou 510640, China; School of Mathematics, South China University of Technology, Guangzhou 510640, China; School of Mathematics, South China University of Technology, Guangzhou 510640, China

**Keywords:** critical point, optimal transport, dynamic network biomarker (DNB), pre-disease stage, sample-perturbed Gaussian graphical model (sPGGM)

## Abstract

Complex disease progression typically involves sudden and non-linear transitions accompanied by devastating effects. Uncovering such critical states or pre-disease stages and discovering dynamic network biomarkers (signaling molecules) is vital for both comprehending disease progression and preventing or delaying disease deterioration. However, the detection of critical points using high-dimensional limited sample data or single-cell data proves notably challenging, as traditional statistical approaches often fail to deliver accurate results. In this study, based on optimal transport theory and Gaussian graphical models, we present an innovative computational framework, the sample-perturbed Gaussian graphical model (sPGGM), designed to analyze disease progression and identify pre-disease stages at the specific sample/cell level. Specifically, by employing population-level optimal transport and Gaussian graphical models, the proposed sPGGM effectively characterizes dynamic differences between the baseline distribution and the perturbed distribution relative to the specific case sample, thus enabling the identification of pre-disease stages and the discovery of signaling molecules during disease progression. The reliability and effectiveness of our method is demonstrated by conducting a simulated dataset and evaluating various data types, including four single-cell datasets, influenza infection data, and six distinct bulk tumour datasets. In comparison with existing single-sample methods, our proposed method exhibits improved capability in pinpointing critical point or pre-disease stages. Moreover, the effectiveness of computational results is highlighted through the analysis of the functional roles of signaling molecules.

## Teaser

A novel sPGGM computational framework detects critical stages and identifies key molecules by quantifying sample-specific/individual perturbations using dynamic network biomarker (DNB), optimal transport and Gaussian graphical models.

## INTRODUCTION

**Figure 1.**
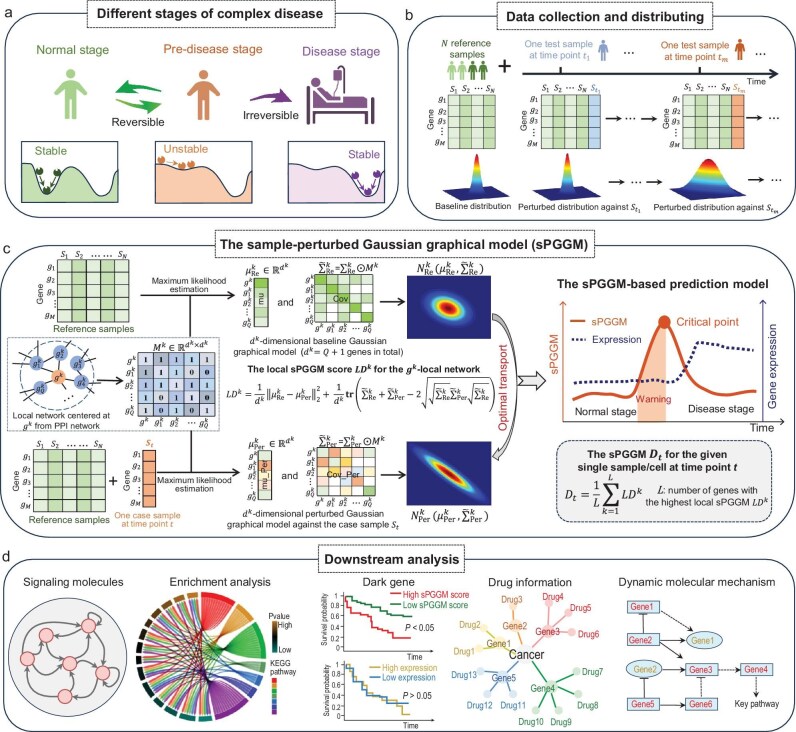
Schematic illustration of sample-perturbed Gaussian graphical model (sPGGM) for identifying pre-disease stages. (a) Disease progression can be classified into three states: the normal stage, pre-disease stage and disease stage, with the pre-disease stage representing a critical threshold just before the onset of disease symptoms. (b) The baseline distribution is fitted from reference samples, whereas the perturbed distribution is derived from mixed samples that combine a specific case sample with the reference group. (c) The proposed sPGGM constructs candidate detection stages at the single-sample level by utilizing a Gaussian graphical model embedded with prior knowledge of the PPI network and quantifies the distributional changes between the baseline and perturbed distributions through the application of optimal transport theory. Then the sPGGM score is used to measure the critical transitions of complex diseases, with a marked increase signaling the pre-disease stage. (d) In downstream analysis, we validate the results by identifying signaling molecules, performing functional analyses, investigating potential molecular regulatory mechanisms, and so on.

Disease progression is inherently dynamic and prone to dramatic shifts over time, often triggered by subtle internal or external disturbances, leading to irreversible and severe consequences. Such a process marked by abrupt critical shifts can typically classified into three phases [1,2]: the normal stage, pre-disease stage and disease stage (Fig. 1a). The normal stage reflects a relatively healthy condition in disease progression, where the system maintains normality and exhibits high stability. The pre-disease stage marks the critical threshold preceding the appearance of disease symptoms [3,4]. That is, as this critical point approaches, the patient often experiences a catastrophic and irreversible transition, commonly resulting in deterioration. In contrast to the reversible normal stage, the irreversible deterioration of disease stage poses a serious threat to the life and health of patients. Therefore, grasping the dynamics of disease progression and unveiling the pre-disease stage plays a key role in facilitating early disease intervention and treatment [5,6]. However, the accurate detection of the pre-deterioration stage or critical point for complex diseases presents a considerable difficulty. There show only minor changes in gene expression patterns and clinical phenotypes between the normal stage and the pre-disease stage. Additionally, challenges such as data noise, patient heterogeneity, limited sample sizes, and model inaccuracies hinder the reliable identification of critical transitions.

The identification of critical transitions in disease progression have increasingly gained attention in recent studies. The computational approach, grounded in flux theory of non-equilibrium dynamical systems, has been devised to estimate various properties of state transitions in the system [7]. Despite its robust theoretical foundation, the high computational complexity of this method makes it challenging to use in large-scale, high-dimensional biological systems [8]. Recently, a new concept of dynamic network biomarkers (DNBs) has been introduced to pinpoint key transitions in complex biological systems [9,10]. Unlike conventional biomarkers that solely assess static molecular activity levels, DNBs can uncover the critical points and potential molecular mechanisms of biological processes. The application of the DNB theoretical framework has shown effectiveness in analyzing critical states of complex diseases like diabetes, cancer and Alzheimer's disease [11–15]. However, existing DNB methods predominantly rely on multiple samples to estimate statistical conditions [9], which constrains their application in biological research due to the challenge of collecting multi-sample data from each time point in practical scenarios. In addition, while these methods mainly aim to detect early critical signals through traditional bulk omics analysis, they still face robustness problem in noisy and heterogeneous single-cell data. In summary, previous methods are limited in their ability to address specific issues in complex diseases and cannot effectively handle challenges such as small sample sizes, highly noisy data, and sample heterogeneity. Therefore, our aim is to design a novel single-sample approach that effectively overcomes these limitations, allowing for the identification of pre-disease stages and the prediction of the important molecules driving disease progression.

Single-cell data has been proven to provide unprecedented insights into the dynamic processes of cellular systems [16,17]. In recent years, there has been growing interest in characterizing transitions at the single-cell level. For instance, methods like MuTrans and QuanTC have been proposed to precisely dissect transition cells from single-cell data [18,19]. BioTIP has been developed to detect critical transition signals from single-cell transcriptomes [20]. Meanwhile, quantifying the critical properties of complex biological systems from a distributional perspective is gaining increasing attention. The multivariate distribution method has been used to detect critical states during complex biological processes [21]. The Gaussian distribution-based model has been proposed to accurately identify critical transitions in disease progression [22]. Additionally, the Kullback–Leibler divergence index has been employed to pinpoint the critical state of cancer by capturing dynamic distributional changes [23]. Such integration of gene expression profiles with distribution-based approaches is vital for more effectively characterizing the dynamic changes within biological systems. In this research, inspired by pioneer works, we propose a new and generalized method called sample-perturbed Gaussian graphical model (sPGGM) based on optimal transport theory and Gaussian graphical models, to identify the critical point or pre-disease stage and discover signaling molecules during disease progression from a sample-specific perspective. Specifically, to reduce irrelevant variables and improve actual biomolecular associations, our proposed sPGGM constructs candidate stages of detection at a single-sample level using a Gaussian graphical model embedded with prior knowledge of the protein-protein interaction network. Then, sPGGM captures the distributional changes between the baseline distribution (fitted from reference samples) and the perturbed distribution (fitted from mixed samples that combine a specific case sample with reference group) through optimal transport [24], and utilize the Wasserstein distance to quantify the relative differences between various detection stages (Fig. 1b and c). The critical properties of complex disease can be unveiled by sPGGM, with significant increases serving as a critical signal for disease prediction due to its sensitivity to distribution shifts and ability to measure the minimal ‘effort’ required to transition from the normal stage to the pre-disease stage. To demonstrate the robustness and effectiveness of sPGGM, we applied it to both simulated data and various real-world disease datasets, including an influenza dataset, two single-cell datasets, and six cancer datasets from the TCGA database: colon adenocarcinoma (COAD), thyroid carcinoma (THCA), kidney clear cell carcinoma (KIRC), uterine corpus endometrial carcinoma (UCEC), kidney renal papillary cell carcinoma (KIRP), and liver hepatocellular carcinoma (LIHC). The results indicate that the proposed sPGGM effectively handles real-world disease data, accurately detects pre-disease stages across various disease categories, and identifies signaling molecules at critical points. Moreover, it exhibits a better performance in capturing critical signals of complex diseases compared to other existing single-sample detection approaches [25–28]. In addition, we conducted functional analysis on the signaling molecules identified by sPGGM, uncovering potential molecular regulatory mechanisms in disease progression and understanding the biochemical basis of disease (Fig. 1d). In brief, our sPGGM provides a new single-sample way to identify the pre-disease state and discover signaling molecules leading to potential disease, which showcases exceptional effectiveness and robustness for both bulk and single-cell data analyses, offering a novel perspective for personalized disease prediction.

## RESULTS

### Performance of the sPGGM based on numerical simulation

**Figure 2.**
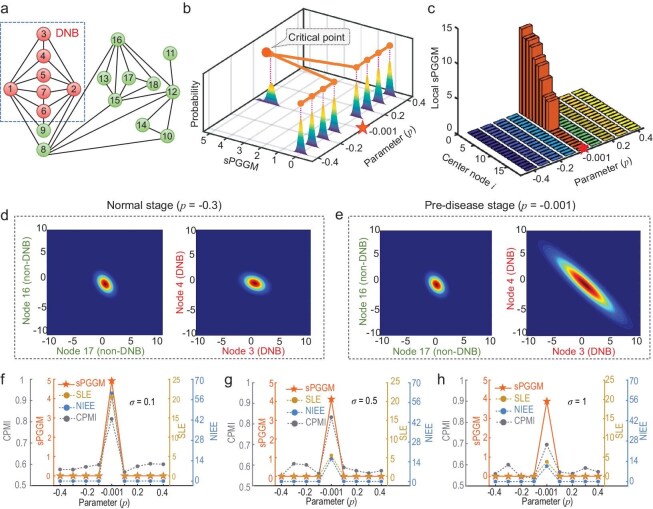
Performance assessment of the sPGGM based on numerical simulation. (a) The numerical simulation is performed using an 18-node graph, constructed from a gene modulated network to depict the relationships between the nodes. (b) The sPGGM curve reveals that a sharp increase in the sPGGM score signals the impending critical transition. (c) The sPGGM landscape shows that the scores for specific local networks with signaling molecules or DNBs exhibit a sharp increase as the system approaches the tipping point. (d, e) The comparison of the multivariate Gaussian distributions for nodes 3 and 4 (DNBs) versus nodes 16 and 17 (non-DNBs) is analyzed between the normal state ($p\ = - 0.3$) and the critical point ($p\ = - 0.001$). (f–h) A comparison of the resilience performance between sPGGM and previous single-sample methods is provided.

To assess the validity of our proposed sPGGM, an 18-node modulated network is used to demonstrate how the algorithm captures critical signals or tipping points (Fig. 2a). Such a modulated network is represented by a framework of stochastic differential equations based on Michaelis-Menten or Hill dynamics, which is commonly used to analyze gene regulation in biological processes, including transcription, translation, and complex nonlinear interactions [29,30]. The network's critical signal is governed by the equation parameter *p*, where $p = 0$ indicates the bifurcation marking the critical point (refer to Section A of the Supplementary Information for further details). Moreover nodes 1 to 7, functioning as signaling molecules or DNBs, are directly influenced by the parameter *p*, while the other nodes remain unaffected by *p* and act as irrelevant molecules. Simulated data is generated by adjusting the parameter *p* from −0.4 to 0.4, illustrating how effectively the sPGGM uncovers the critical transition near the bifurcation.

It can be seen from Fig. 2b that a notable rise in the sPGGM score signals the impending critical state as the system nears the bifurcation point ($p = 0$), while the score remains stable and low when the system is distant from the tipping point. Moreover, to reveal the specific dynamics of each node and pinpoint signaling molecules throughout the progression, we show the evolution of local sPGGM landscapes across different nodes in Fig. 2c. As the system is away from the tipping point, the sPGGM score for all local networks remains uniformly low, but a notable spike in the sPGGM score occurs in specific local networks containing DNBs when nearing the critical point. Additionally, Fig. 2d and e illustrates the transport of distributions from normal states to the critical point. As the system approaches the tipping point, the multivariate Gaussian distribution of signaling molecules becomes more divergent and fluctuates significantly, indicating a substantial rise in their variance. To demonstrate the resilience of sPGGM, we conducted a comparative analysis of sPGGM and other existing single-sample methods on samples subjected to varying levels of noise perturbation (Fig. 2f–h), highlighting our proposed method's superior sensitivity and clarity in detecting critical signals. As the noise level increases, our sPGGM method demonstrates enhanced robustness and efficacy when subjected to high noise levels (Fig. 2f–h and Fig. S1). The simulation results show that the sPGGM effectively and accurately detects critical transitions. Besides, our proposed sPGGM can pinpoint signaling molecules and shed light on the key changes during system progression.

### Identifying pre-disease stages for individual influenza infection

**Figure 3.**
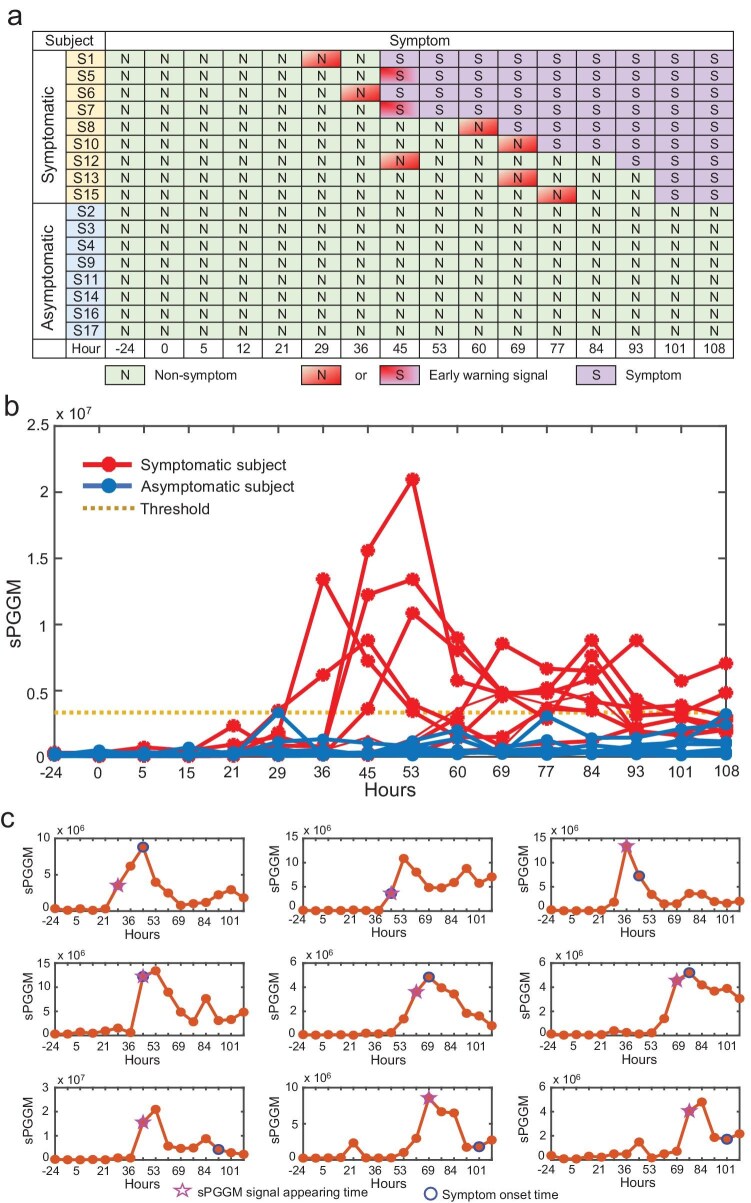
Identification of pre-disease stages for influenza infection based on sPGGM. (a) A temporal chart detailing the onset of influenza symptoms and the pre-disease stages determined by sPGGM for all participants. (b) The sPGGM curves for all 17 subjects are shown, with the red curve representing the nine symptomatic participants and the blue curve depicting the eight asymptomatic participants. (c) The curves for sample-specific sPGGM score of nine symptomatic individuals are displayed, where the blue circle indicates the onset of influenza symptoms (as clinically observed), and the pink box marks critical signals identified by sPGGM.

In this research, we utilized the sPGGM to analyze the time-series dataset related to influenza infection. This dataset consists of samples from 17 volunteers infected with the Wisconsin/H3N2 virus via intranasal administration, with gene expression data collected at 16 time points over a 132-hour span (−24 to 108 hours) (Fig. 3a). Among them, 9 volunteers (subjects 1, 5, 6, 7, 8, 10, 12, 13, and 15) with severe influenza-like symptoms were classified as the symptomatic group, while the remaining 8 volunteers showing no clinical symptoms were categorized as the asymptomatic group. For each participant, the gene expression data from the first four time points were considered as a reference group, indicating their relatively healthy state. The sample-specific sPGGM score (denoted as ${D}_t$ in Eq. (S35)) was calculated for each of 17 participants by the algorithm detailed in the Materials and Methods section. A rapid increase in the sPGGM score acts as an early indicator of disease onset, particularly signaling the moment when clinical symptoms begin to manifest. Fig. 3b depicts the sPGGM score for all participants across each time point. The symptomatic group (indicated by red curves) show a marked increase in the sPGGM score prior to the appearance of symptoms, providing early signs of imminent critical transitions. In contrast, the asymptomatic group (shown by blue curves) exhibit consistent sPGGM scores with no significant changes. Moreover, Fig. 3c depicts the sPGGM score tailored for each of the nine symptomatic participants, highlighting the pre-disease stage that precedes the emergence of clinical symptoms in each case. Consequently, the identification of the pre-disease stages for each participant validates the effectiveness of our sPGGM from a sample-specific viewpoint.

#### Identifying pre-disease stages for cancer progression

**Figure 4.**
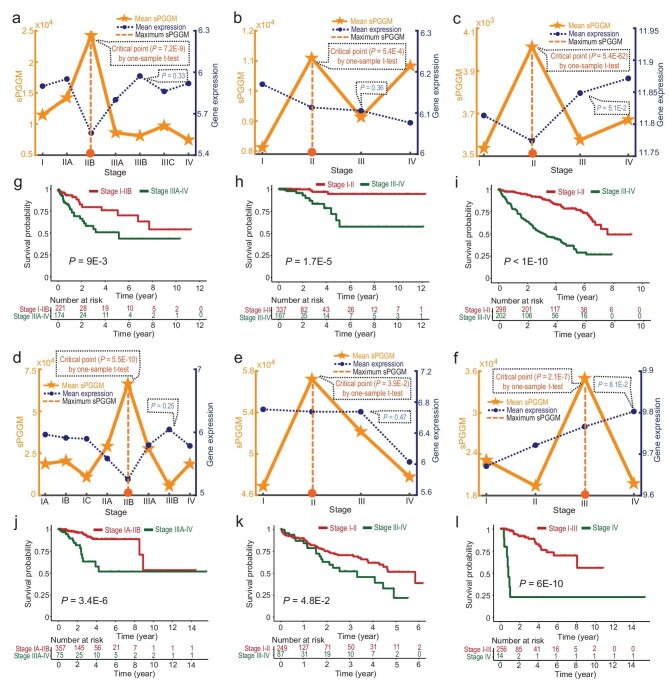
Identification of pre-disease stages for cancer progression based on sPGGM. The dynamic behavior of sPGGM and gene expression was evaluated across six tumour types: (a) COAD, (b) THCA, (c) KIRC, (d) UCEC, (e) LIHC, and (f) KIRP. The significant rise in the sPGGM score indicates an upcoming critical transition before disease deterioration. Survival durations before and after the critical stage was analyzed for the following tumour types: (g) COAD, (h) THCA, (i) KIRC, (j) UCEC, (k) LIHC, and (l) KIRP. Patients experience significantly longer survival times before reaching the critical point compared to after it.

To determine how well the proposed sPGGM unveils pre-disease stages of cancer progression, we applied this method to six tumour datasets (COAD, THCA, KIRC, UCEC, KIRP, and LIHC) sourced from the TCGA database. Using adjacent non-tumour samples as the reference group, we determined the sample-specific sPGGM score (as outlined in Eq. (S35)) for each individual case. The mean sPGGM score at each stage was applied as a quantitative indicator to evaluate the pre-disease state. The analytical findings revealed that the pre-disease stage was identified as stage II for THCA, KIRC, and LIHC, stage III for KIRP, and stage IIB for COAD and UCEC (Fig. 4a–f). In the COAD dataset, a significant shift ($P\ = \ 7.2{\mathrm{E}} - 9$) in the sPGGM score was observed around stage IIB (Fig. 4a), signaling the onset of lymph node metastasis at stages IIIA–IIIB [31]. For the THCA dataset, it is seen from Fig. 4b that the sPGGM score reaches its highest point at stage II ($P\ = \ 5.4{\mathrm{E}} - 4$), indicating an approaching critical shift. The literature reveals that stage III involves the sternothyroid muscle or nearby thyroid-related soft tissues, along with metastasis to regional lymph nodes [32]. When applied to the KIRC dataset, as illustrated in Fig. 4c, a notable increase ($P\ = \ 5.4{\mathrm{E}} - 62)$ in the sPGGM score from stages I to II points to a critical deterioration event, that is, stage III is characterized by a rapid escalation of lipid levels around the kidney and tumour invasion into the renal vein [33]. In the UCEC dataset, there is a substantial increase ($P = 5.5{\mathrm{E}} - 10$) in the sPGGM score between stages IIA and IIB (Fig. 4d), implying the occurrence of tumour extension into surrounding tissues or metastasis to lymph nodes after stage IIB [34]. For the LIHC dataset, the sPGGM score shows a sudden rise ($P = 3.9{\mathrm{E}} - 2$) during stage II (Fig. 4e), after which direct invasion of nearby organs appears [35]. When applied to the KIRP dataset, the sPGGM score increased drastically before stage III ($P = 2.1{\mathrm{E}} - 7$), signaling that distant metastasis generally occurs at stage IV (Fig. 4f) [36]. Conversely, as depicted by the dark blue curve in Fig. 4a–f, the expression levels of highly expressed genes from specific samples does not effectively indicate a critical transition from the perspective of both accuracy and signal significance. Furthermore, compared to four other existing single-sample methods [25–28] (see Table 1 and Figs S2–S4), our proposed sPGGM demonstrates improved performance in identifying pre-disease stages throughout the progression of the disease.

**Table 1.** Comparison of the performance among different single-sample detection methods

Dataset	sPGGM	L-DNB	SLE	CPMI	NIEE
COAD	Stage IIB (*P* = 7.2E-9)	Stage IIIC (*P* = 9.6E-11)	Stage IIB (*P* = 7.7E-9)	Stage IIB (*P* = 4.5E-5)	Stage IIIC (*P* = 1.2E-50)
THCA	Stage II (*P* = 5.4E-4)	Stage III (*P* = 1.4E-2)	Stage III (*P* = 1.5E-2)	Stage III (*P* = 2.1E-4)	None
KIRC	Stage II (*P* = 5.4E-62)	Stage II (*P* = 3.2E-2)	None	Stage III (*P* = 4.2E-2)	Stage IV (*P* = 1.1E-13)
UCEC	Stage IIB (*P* = 5.5E-10)	Stage IIB (*P* = 6.5E-257)	Stage IIB (*P* = 6.4E-4)	Stage IIB (*P* = 6.8E-84)	Stage IIIA (*P* = 3.2E-6)
LIHC	Stage II (*P* = 3.9E-2)	None	None	Stage IV (*P* = 2.6E-2)	Stage III (*P* = 3.7E-57)
KIRP	Stage III (*P* = 2.1E-7)	Stage III (*P* = 1.9E-11)	Stage III (*P* = 1.2E-308)	Stage II (*P* = 4.6E-2)	Stage III (*P* = 3.1E-3)
T-cell exhaustion	Stage 5 (*P* = 1.2E-129)	Stage 7 (*P* = 1.7E-2)	Stage 7 (*P* = 2.4E-2)	Stage 5 (*P* = 1.1E-2)	None
GABAergic interneurons	D54 (*P* = 1.8E-2)	None	None	None	D125 (*P* = 4.7E-4)

None: represents the inability to detect the critical signal.

To verify the determined pre-disease state, we utilized the Kaplan–Meier (log-rank) method to conduct a prognostic survival analysis on clinical samples taken from before and after the critical point. It can be observed from Fig. 4g–l that there shows a significant difference in prognosis between patients diagnosed before and after the critical stage, with *p* values below 0.05, indicating that those treated before critical transition have higher survival rates and longer survival times. Therefore, the sPGGM score can effectively signal the pre-disease states related to survival time before disease deterioration, which facilitates prompt medical intervention and follow-up care.

#### Functional analysis of the signaling molecules involved in cancer progression

**Figure 5.**
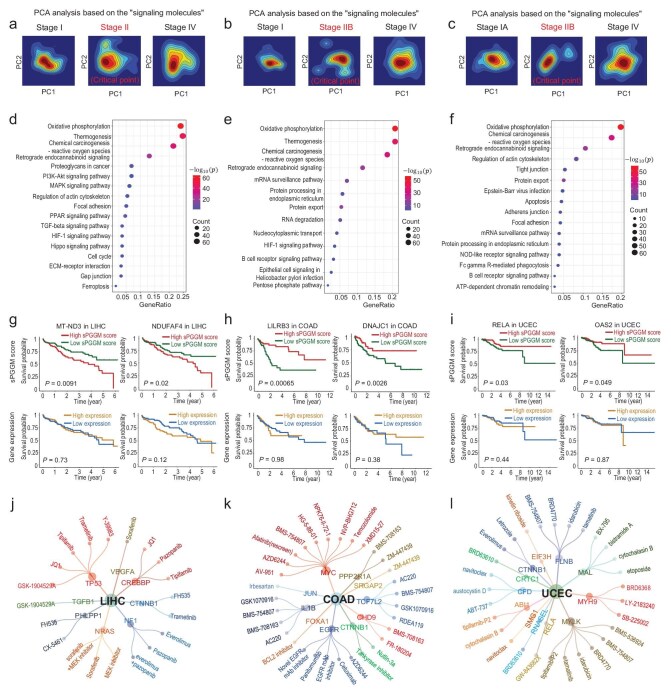
Functional analysis of the signaling molecules implicated in cancer development. PCA-based visualizations of the optimal transport map transitioning from the normal to abnormal states for three tumour types: (a) LIHC, (b) COAD, and (c) UCEC. The KEGG pathway enrichment analysis of signaling molecules in three tumour types: (d) LIHC, (e) COAD, and (f) UCEC. The results demonstrate that signaling molecules are mainly enriched in cancer-associated pathways. The survival analysis of ‘dark molecule’ (non-differential genes sensitive to the sPGGM score) for three tumour types: (g) LIHC, (h) COAD, and (i) UCEC. This survival analysis, based on the local sPGGM score rather than gene expression values, proves effective in prognosis and successfully distinguishes significant differences in survival times. The key signaling molecules targeted by specific cancer-related drugs identified for three tumour types: (j) LIHC, (k) COAD, and (l) UCEC.

In addition to detecting the early pre-disease stages of tumour progression, we also conduct a functional analysis of signaling molecules (the top 5% of genes exhibiting the highest sPGGM score) to gain insights into their role in disease development. The transport map derived from the normal to abnormal state via sPGGM enables us to describe the disease progression using Gaussian graphical distributions. In this study, we utilize principal component analysis (PCA) [37] to demonstrate the main transformation processes in the distribution of signaling molecules at different stages of the disease. As illustrated in Fig. 5a–c, the distribution transport process for three tumour datasets (LIHC, COAD and UCEC) reveals that the distribution becomes more concentrated as it moves away from critical points and gradually disperses as it nears them. The temporal evolution of the distribution transport process across all stages is presented in Fig. S5. This indicates that the sPGGM effectively captures these distribution state changes and identifies critical transitions across various diseases. Furthermore, functional enrichment analysis of the identified signaling molecules shows a significant enrichment in cancer-related pathways, including oxidative phosphorylation [38], chemical carcinogenesis—reactive oxygen species [39], and PI3K-Akt signaling pathway [40] (Fig. 5d–f). An additional functional analysis of both ‘dark molecule’ (non-differential genes sensitive to the sPGGM score) and differentially expressed genes (DEGs) among signaling molecules is given in Fig. S6. Additionally, we also discovered that certain ‘dark molecules’ involved in cancer-related pathways are essential for disease progression and serve as effective prognostic indicators, not at the gene expression level but at the sPGGM score level (Fig. 5g–i). Therefore, our sPGGM-based method can be viewed as a valuable complement to traditional differential expression analysis, helping to identify new biomarkers, drug targets, and prognostic indicators from a network-level perspective (since the sPGGM score is derived from network-based computations) rather than focusing solely on the gene level. Moreover, to enhance the validation of ‘dark molecules’ as prognostic indicators, Figs S7 and S8 present a comparison of survival analysis among the ‘dark molecule’, the top 5% most significant DEGs, and randomly selected genes of the same size. Besides, it can be seen from Fig. 5j–l that key signaling molecules targeted by specific cancer-related drugs can be identified based on the iGMDR database [41], which provides drug targets and effective drugs for early therapeutic intervention in patients with specific cancer.

#### Identifying pre-disease stages for complex diseases at single-cell level

**Figure 6.**
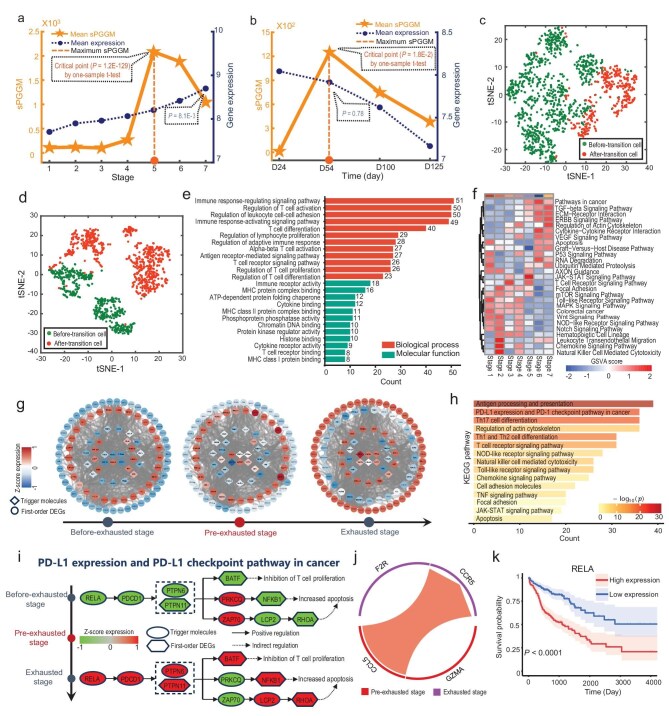
Identifying pre-disease stages for complex diseases at the single-cell level. The dynamic behavior of sPGGM and gene expression analyzed for (a) CD8+ T-cell exhaustion dataset and (b) GABAergic interneurons dataset. The cell clustering results based on t-distributed stochastic neighbor embedding (t-SNE) of the signaling molecules for (c) CD8+ T-cell exhaustion dataset and (d) GABAergic interneuron dataset. (e) Gene ontology (GO) analysis for functional enrichment indicated that the targeted signaling molecules are enriched in biological processes related to CD8+ T-cell exhaustion. (f) Functional analysis using GSVA indicates that targeted signaling molecules have distinct roles in the progression of CD8+ T-cell exhaustion. (g) The dynamic evolution of the regulatory network constructed from targeted signaling molecules and their neighboring differentially expressed genes (DEGs) was explored during the CD8+ T-cell exhaustion process. (h) KEGG pathway enrichment analysis was carried out for the first-order DEGs. (i) The functional analysis of targeted signaling molecules and first-order DEGs revealed the signaling mechanisms associated with the CD8+ T-cell exhaustion in the PD-L1 expression and PD-1 checkpoint pathway in cancer. (j) Cellular communication between the pre-exhausted CD8+ T-cell subset (stage 5) and the exhausted CD8+ T-cell subset (stage 7) occurs through *CCL5*_*CCR5* and *GZMA*_*F2R* receptor-ligand interactions. (k) Survival analysis of *RELA* based on the TCGA-COAD dataset.

To gain deeper insights into the pre-disease stages of complex diseases at the single-cell resolution, we applied the proposed sPGGM to two disease-associated single-cell data: CD8+ T-cell exhaustion dataset and GABAergic interneurons dataset. For the CD8+ T-cell exhaustion dataset, the brown-yellow curve in Fig. 6a indicates a marked increase in the sPGGM score at stage 5 ($P\ = \ 1.2{\mathrm{E}} - 129$), signaling an early warning of the impending critical transition for the cell subpopulation which began to exhibit exhaustion characteristics thereafter [42]. When applied to the GABAergic interneurons dataset, it is seen from Fig. 6b that the sPGGM score demonstrates a significant rise from day 24 to day 54 ($P\ = \ 1.8{\mathrm{E}} - 2$), after which there is a transition from neurogenesis to gliogenesis, with certain genes involved in astrocyte function [43]. In contrast, the dark blue curve shown in Fig. 6a and b reveals that the expression levels of highly expressed genes do not adequately signify a critical transition in terms of accuracy and signal significance. As illustrated in Fig. S9A, for the T-cell exhaustion dataset, sPGGM, MuTrans, and BioTIP detects the critical transition with significant increases, with the sPGGM providing an earlier warning signal. When applied to the GABAergic interneurons dataset, it is seen from Fig. S9B that sPGGM, MuTrans, QuanTC, and BioTIP consistently indicate the critical state at day 54, with the sPGGM showing the most statistically significant signal. Moreover, the sPGGM is a sample-perturbed critical point detection model, meaning that it can detect critical signals at the specific sample/cell level when given a set of reference samples. Therefore, the sPGGM has its own specific advantages in analyzing critical states. At the identified critical state, the top 5% of genes showing the highest local sPGGM value were chosen as signaling molecules for further analyses of functional roles and biological processes. It is seen from Fig. 6c–d that the cell clustering results based on signaling molecules clearly differentiate the stages before and after the critical transition, specifically around stage 5 for the CD8+ T-cell exhaustion dataset and day 54 for the GABAergic interneurons dataset.

Transcription factors (TFs) have been shown to play a pivotal role in regulating CD8+ T-cell exhaustion in colorectal cancer (CRC) by controlling the expression of related target genes [44]. To investigate the mechanism of signaling molecules, we conducted an analysis to explore the functions of signaling molecules regulated by upstream transcription factors. In this study, 67% of signaling molecules were found to be regulated by TFs (Fig. S10), suggesting their potential role in mediating CD8+ T-cell exhaustion in CRC. Moreover, we performed GO enrichment analysis on targeted signaling molecules. As shown in Fig. 6e, they are significantly enriched in biological processes associated with CD8+ T-cell exhaustion, such as the immune response-regulating signaling pathway, regulation of T-cell activation, and regulation of leukocyte cell-cell adhesion. Additionally, from a molecular function standpoint, they are primarily enriched in MHC protein complex binding, cytokine binding, and T-cell receptor binding, with disruptions in these functions directly impairing T-cell activation, reducing anti-tumour immunity, and celebrating T-cell exhaustion [45]. GSVA analysis of the targeted signaling molecules was conducted to further explore the dynamic changes in biological functions and pathways throughout the CD8+ T-cell exhaustion process [46]. As illustrated in Fig. 6f, the GSVA score for pathways such as Pathways in cancer, TGF-beta signaling pathway, ECM-receptor interaction, ERBB signaling pathway, and Regulation of actin cytoskeleton exhibits an upward trend as the exhaustion process advances. The upregulation of these pathways may promote the formation of an immunosuppressive microenvironment, thereby inhibiting CD8+ T-cell function and ultimately affecting the anti-tumour immune response.

To further uncover the molecular mechanisms behind tumour progression at the network level, functional analysis was performed on the PPI subnetworks of targeted signaling molecules, constructed from these molecules and their neighboring differentially expressed genes (DEGs) within the PPI network. As illustrated in Fig. 6g, a distinct shift in gene expression patterns within the networks emerges after the pre-exhaustion stage, with significant changes in the expression levels of targeted signaling molecules and their first-order DEGs, underscoring the crucial role of these reversed genes in CD8+ T-cell exhaustion. Moreover, the KEGG enrichment analysis shows that they are significantly enriched in cancer immunology-related pathways, such as the antigen processing and presentation, the PD-L1 expression and PD-1 checkpoint pathway in cancer, and the T-cell receptor signaling pathway (Fig. 6h). Notably, the PD-L1 expression and PD-1 checkpoint pathway in cancer represents a particularly significant immunological pathway related to CD8+ T-cell exhaustion. It is observed that the upregulation of *RELA*, acting as a targeted signaling molecule regulated by transcription factors (TFs), drives the high expression of *PDCD1* and *PTPN6/PTPN11* (Fig. 6i), which may play a critical role in CD8+ T-cell exhaustion. Specifically, the upstream signaling molecule *RELA* can promote the high expression of *PDCD1* (PD-1), an inhibitory receptor crucial for CD8+ T-cell exhaustion [47]. *PDCD1* subsequently drives the high expression of *PTPN6* and *PTPN11*, which mainly negatively regulate TCR signaling through dephosphorylation, leading to a reduction in T-cell activity [48]. The activation of *PTPN6/PTPN11* triggers several key downstream signaling pathways: first, *PTPN6/PTPN11* inhibit T-cell proliferation by activating *BATF* [49]. Second, they suppress the expression of *PRKCQ*, which reduces the activation of the NF-κB signaling pathway, leading to decreased T-cell survival signals and increased apoptosis of T-cells [50]. Additionally, they negatively regulate TCR signaling by inducing low expression of *ZAP70*, a critical molecule in T-cell receptor signaling, thereby weakening TCR-mediated cell activation [51]. Consequently, the low expression of *ZAP70* activates the high expression of *LCP2* and *RHOA* primarily involved in cytoskeletal remodelling and regulation. Its upregulation affects T-cell migration and the formation of immune synapses by modulating the cytoskeleton, thereby diminishing T-cell activation and further promoting T-cell apoptosis [52]. Thus, our findings suggest that the upregulation of *RELA* drives the high expression of key molecules such as *PDCD1* and *PTPN6/PTPN11* (Fig. S11), which may activate downstream signaling pathways that mediate CD8+ T-cell exhaustion. In terms of intercellular communication, the pre-exhausted CD8+ T-cell subset (stage 5) transmits a robust exhaustion signal to the exhausted CD8+ T-cell subset (stage 7), with *CCL5*_*CCR5* and *GZMA*_*F2R* receptor-ligand interactions positively regulating this process (Fig. 6j), thereby promoting CD8+ T-cell exhaustion. Additionally, the prognosis analysis results indicate that high *RELA* expression in tumour tissues is associated with poor overall survival (Fig. 6k).

## DISCUSSION

Identifying the pre-disease stages of complex diseases is crucial for preventing or delaying disease deterioration. However, traditional methods are not well-suited to capture the dynamics of disease progression and often fail to identify critical transitions in real biological datasets characterized by high data noise, patient heterogeneity, and small sample sizes. To address this challenge, based on our recently proposed DNB theory [53], along with the concepts of population-level optimal transport and Gaussian graphical models [24], we present a robust computational method called the sPGGM, which effectively reveals critical points or pre-disease stages and identifies signaling molecules involved in critical transitions from a sample-specific perspective. Our proposed sPGGM has been validated with simulated data and applied to the analysis of both scRNA-seq and bulk sequencing data across various diseases, including four single-cell datasets, influenza infection data, and six distinct tumour datasets (COAD, THCA, KIRC, UCEC, KIRP, and LIHC). The accurate prediction of pre-disease stages for these complex diseases at the specific sample/cell level highlights that our method is a valuable tool for health assessment and personalized precision medicine. Additionally, the strong performance of the sPGGM in identifying disease-related critical states was proven through comparisons with previous single-sample approaches on both single-cell and bulk data.

The advantages of our proposed sPGGM can be briefly summarized as follows. First, being a distribution-based model, the sPGGM exhibits a strong robustness and stability, as shown by its effective performance across bulk data of small sample sizes and high-noise single-cell data. Second, by introducing Gaussian graphical optimal transport to measure the dynamic differences between baseline and sample-perturbed distributions, the sPGGM outperforms existing single-sample methods in identifying pre-disease stages during disease progression. Third, given a set of reference samples, the sPGGM not only identifies critical signals toward a deteriorated stage at the sample-specific level but also highlights key signaling molecules associated with crucial biological processes, offering significant advancements in disease pathology analysis and personalized precision medicine. In particular, the trend of the signal curve, i.e. a sudden increase as it approaches the critical point, indicates that our proposed method is effective when the sizes of the reference samples fall within a specified range (Figs S12 and S13). Fourth, unlike traditional techniques that rely on differential equations for simulations, the sPGGM emphasizes data-driven insights by directly extracting information from the data, without the need for predefined parameters. Moreover, the sPGGM can demonstrate its scalability and effectiveness for analyzing large single-cell RNA-seq datasets (over 2 million cells across five time points and 356,213 cells from six age groups) (Fig. S14). However, a limitation of the sPGGM is its reliance on undirected networks, which overlook causal relationships between nodes, presenting a potential area for improvement in our future research.

## METHODS

### Theoretical background

Disease progression frequently exhibits abrupt shifts in temporal patterns and can be described as a time-varying nonlinear process in the context of dynamical systems, where a sudden state deterioration signifies a qualitative transition at a bifurcation point [54]. The dynamic progression of diseases generally is defined by three states (see Fig. 1a): (i) a normal stage characterized by minimal fluctuation and strong resilience; (ii) a pre-disease stage marked by inherent instability and considerable complexity, indicating a critical transition toward a disease deterioration state; and (iii) a subsequent disease stage associated with the onset or worsening of the disease. The key to detect pre-disease stages or critical points lies in developing an index that quantitatively measures dynamical changes in the state of disease systems. However, the difficulty in distinguishing between the normal stage and the pre-disease stage becomes more evident when compared to the disease state. Therefore, traditional statistical techniques may struggle to differentiate the pre-disease stage.

Based on our recently proposed theoretical concept of DNB [9,54], as the system nears a critical point, a set of molecules (DNB variables) with strong correlations and large fluctuations emerges, signaling an impending critical state transition from a network-level perspective. It is evident that the state shift or phase of a system can be characterized by a dynamic change in both the multivariate distribution and molecular associations of DNB members. Therefore, by applying the Gaussian graphical model and optimal transport theory, we introduce the sPGGM to detect critical signals that mark the key transition from the normal stage to the disease stage, which identifies the pre-disease stage from a sample-specific perspective and addresses challenges such as small sample sizes, high noise levels, and sample heterogeneity. In our study, the Gaussian graphical model is defined by a graph structure paired with a Gaussian distribution, enabling the graph to depict the dependencies among molecules within the multivariate Gaussian distribution [55–57]. A comprehensive description of the Gaussian graphical model employed in our analysis is introduced in Section N of the online Supplementary Information.

### A quantitative approach to identify the pre-disease stage based on the sPGGM

Using a set of reference samples/cells obtained from a relatively healthy condition, the proposed sPGGM was employed to identify the pre-disease stage or critical state from a sample-specific/cell-specific perspective. The details of this process are provided in Section N within the online Supplementary Information.

## Supplementary Material

nwaf189_Supplemental_File

## Data Availability

Nine real datasets were employed in this study, which included the influenza infection dataset (GSE30550), GABAergic interneurons dataset (GSE93593) and CD8+ T-cell exhaustion dataset (GSE108989) sourced from the GEO database (http://www.ncbi.nlm.nih.gov/geo/), and COAD, THCA, KIRC, UCEC, KIRP, and LIHC datasets obtained from the TCGA database (http://cancergenome.nih.gov). The source code of the algorithm and related data are available at https://github.com/Junxian-Li-0/sPGGM_project.
