# Supplementary material for: sPGGM: a sample-perturbed Gaussian graphical model for identifying pre-disease stages and signaling molecules of disease progression
Source: Natl Sci Rev. 2025 May 14;12(8):nwaf189. doi: 10.1093/nsr/nwaf189 (PMC12236331; doi:10.1093/nsr/nwaf189)
Supplement: nwaf189_Supplemental_File [file nwaf189_supplemental_file.docx]

**Supplementary Information: a sample-perturbed Gaussian graphical model (sPGGM) for identifying pre-disease stages and signaling molecules of disease progression**

**Contents**

**A.** **Overview of dynamic systems for numerical analysis**  S2

**B.** **Advantages of sPGGM compared to L-DNB method**  S4

**C.** **Performance comparison of various single-sample detection methods**  S5

**D.** **Description of stage transport map for the distribution of signaling molecules**  S6

**E.** **Functional analysis of both dark molecule and DEG among signaling molecule**  S7

**F.** **Performance comparison of sPGGM and transition characterization methods** S11

**G.** **Functional analysis of the CD8+ T-cell exhaustion data** S12

**H.** **Performance of sPGGM across different reference sample size** S13

**I.** **Performance of sPGGM for analysing large single-cell datasets** S14

**J.** **Derivation and proof of the 2-Wasserstein distance for Gaussian measures** S15

**K.** **Performance of sPGGM at different PPI confidence thresholds** S17

**L.** **Comprehensive explanation for focusing on 1st-order interactions** S18

**M.** **Description of unveiling the pre-disease stage** S20

**N.** **Materials and Methods** S20

**O.** **Description of the nine real biological datasets** S24

1. **Overview of dynamic systems for numerical analysis**

To highlight the effectiveness of our sPGGM, we carried out a numerical simulation using an 18-node regulatory network, illustrated in Figure 2a of the main text. Such a modulated network is represented by a framework of stochastic differential equations based on Michaelis-Menten or Hill dynamics, which is commonly used to analyse gene regulation in biological processes, including transcription, translation, and complex nonlinear interactions [1,2]. Specifically, this 18-node modulated network can be characterized by the following set of 18 differential equations.

(S2)

where $p$ is a scalar parameter, and $\zeta_{i}(t)$ (*i* = 1, 2, $\cdots$, 18) denotes Gaussian noise with a mean of zero and covariances given by $k_{ij}=\mathrm{Cov}(\zeta_{i},\zeta_{j})$. The mRNA-i concentrations are described by $z_{i}(t)$ (*i* = 1, 2, $\cdots$, 18). In Eq.(S1), the degradation rates of mRNAs are represented as

$$R\text{=}\left(\frac{(4+4|p|)}{15}\text{,}\frac{(8+2|p|)}{15}\text{,1,}\frac{6}{5}\text{,}\frac{7}{5}\text{,}\frac{8}{5}\text{,}\frac{9}{5}\text{,2,}\frac{11}{5}\text{,}\frac{12}{5}\text{,}\frac{13}{10}\text{,}\frac{14}{10}\text{,5,}\frac{16}{10}\text{,}\frac{7}{2}\text{,}\frac{7}{2}\text{,}\frac{19}{5}\text{,4}\right).$$

$\bar{Z}=(\bar{z}_{1},\bar{z}_{2},\ldots,\bar{z}_{18})=(0,0,\ldots,0)$ represents the stable equilibrium point of the dynamic system outlined in Eq. (S2). By applying the Euler method, Eq. (S2) can be transformed into a corresponding set of discrete equations using a small time step of $\Delta t$.

(S3)

Where $Z(k)$ refers to the vector $Z(t)$ at the time instant $k\Delta t$. The Jacobian matrix for Eq. (S3) is indicated as $J=\left.\frac{\partial f(Z(k);p)}{\partial Z}\right|_{Z=\bar{Z}}$, with

$J=e^{\Delta t\bullet A}$. (S4)

Setting $\Delta t=1$ allows for the derivation of eight distinct eigenvalues from Eq. (S4). The leading eigenvalue satisfies the condition $0.68^{|p|}$→ 1 when $p$→ 0. This behavior indicates that the dominant eigenvalue of the differential system described by Eq. (S2) steadily tends toward 0 from the negative side $p$→ 0. As a result, the equilibrium point $\bar{Z}$ is considered stable if $p\in(0, 1]$. This specific parameter$p=0$ represents the point of bifurcation, which indicates a qualitative change in the system.

**B.** **Advantages of** **sPGGM** **compared to L-DNB method**

Compared to the L-DNB method, our sPGGM has the following two advantages: (1) Advantage of capturing nonlinear correlations: The L-DNB method mainly relies on linear correlation metrics (PCC) to infer gene relationships and identify critical states. However, from a dynamical systems perspective, biological systems are inherently complex and nonlinear [3,4], making it challenging for linear methods like PCC to fully capture their intricate interactions. In contrast, our sPGGM method leverages nonlinear approaches, such as the Wasserstein distance (Equation 6 in the main manuscript), to uncover latent nonlinear dependencies within multivariate Gaussian distributions, thereby enhancing its capability to detect critical transitions during disease progression. (2) Advantage of quantifying "distributional criticality": The L-DNB method primarily relies on characterization directly derived from sample data, making it more vulnerable to noise and outliers. A key improvement of our sPGGM approach lies in the integration of both the optimal transport theory and Gaussian graphical models, which utilizes the probabilistic principles of population-level distributions to effectively mitigates the impact of noisy high-dimensional data and enhance the detection of critical states. Therefore, by incorporating the statistical properties of distributions, our sPGGM improves robustness and provides a more stable identification of pre-deterioration states. Therefore, based on advantage of capturing nonlinear correlations and quantifying "distributional criticality", our sPGGM demonstrates better robustness performance over L-DNB (as shown in Figure S1).


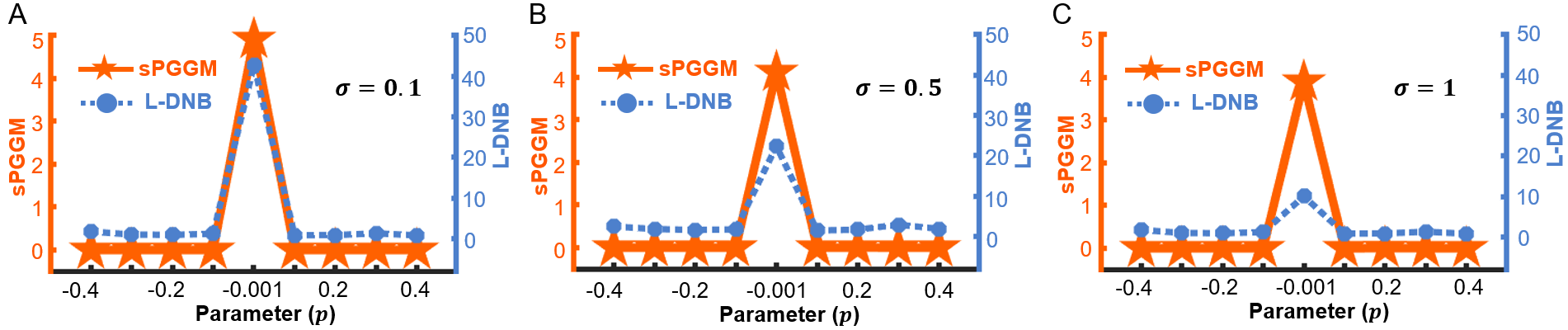


Figure S1. A comparison of the performance between the sPGGM and L-DNB method at different noise intensities: (A) noise level $\sigma=$ 0.1, (B) noise level $\sigma=$ 0.5, and (C) noise level $\sigma=$ 1. As the noise level increases, our sPGGM method demonstrates enhanced robustness and efficacy when subjected to high noise levels.

**Table S1. Comparison of the performance between sPGGM and L-DNB on various real datasets**

| Dataset | COAD | KIRC | KIRP | LIHC |
| --- | --- | --- | --- | --- |
| sPGGM | Stage IIB  (*P* = 7.2E-9) | Stage II  (*P* = 5.4E-62) | Stage III  (*P* = 2.1E-7) | Stage II  (*P* = 3.9E-2) |
| L-DNB | Stage IIIC  (*P* = 9.6E-11) | Stage II  (*P* = 3.2E-2) | Stage III  (*P* = 1.9E-11) | None |
| Dataset | **THCA** | **UCEC** | **T-cell**  **exhaustion dataset** | **GABAergic interneurons dataset** |
| sPGGM | Stage II  (*P* = 5.4E-4) | Stage IIB  (*P* = 5.5E-10) | Stage 5  (*P* = 1.2E-129) | D54  (*P* = 1.8E-2) |
| L-DNB | Stage III  (*P* = 1.4E-2) | Stage IIB  (*P* = 6.5E-257) | Stage 7  (*P* = 1.7E-2) | None |

**C.** **Performance comparison of** **various single-sample detection methods**

Many single-sample methods, such as landscape dynamic network biomarker (LDNB) [5], single-sample landscape entropy (SLE) [6], comprehensive neighbourhood-based perturbed mutual information (CPMI) [7], and network information entropy of edges (NIEE) [8], have been applied to assess the criticality of complex diseases based on individual samples or cells. To showcase the advantages of our proposed sPGGM, we conducted a comparison using six cancer datasets from the TCGA database (COAD, THCA, KIRC, UCEC, KIRP, and LIHC) alongside two single-cell datasets (GABAergic interneurons and T-cell exhaustion data). As demonstrated in Figures S2-S4 and Table 1, our approach yields more significant P-values at critical points and identifies pre-disease states that better correspond to biological significance, highlighting its enhanced performance to detect critical signals during disease progression.


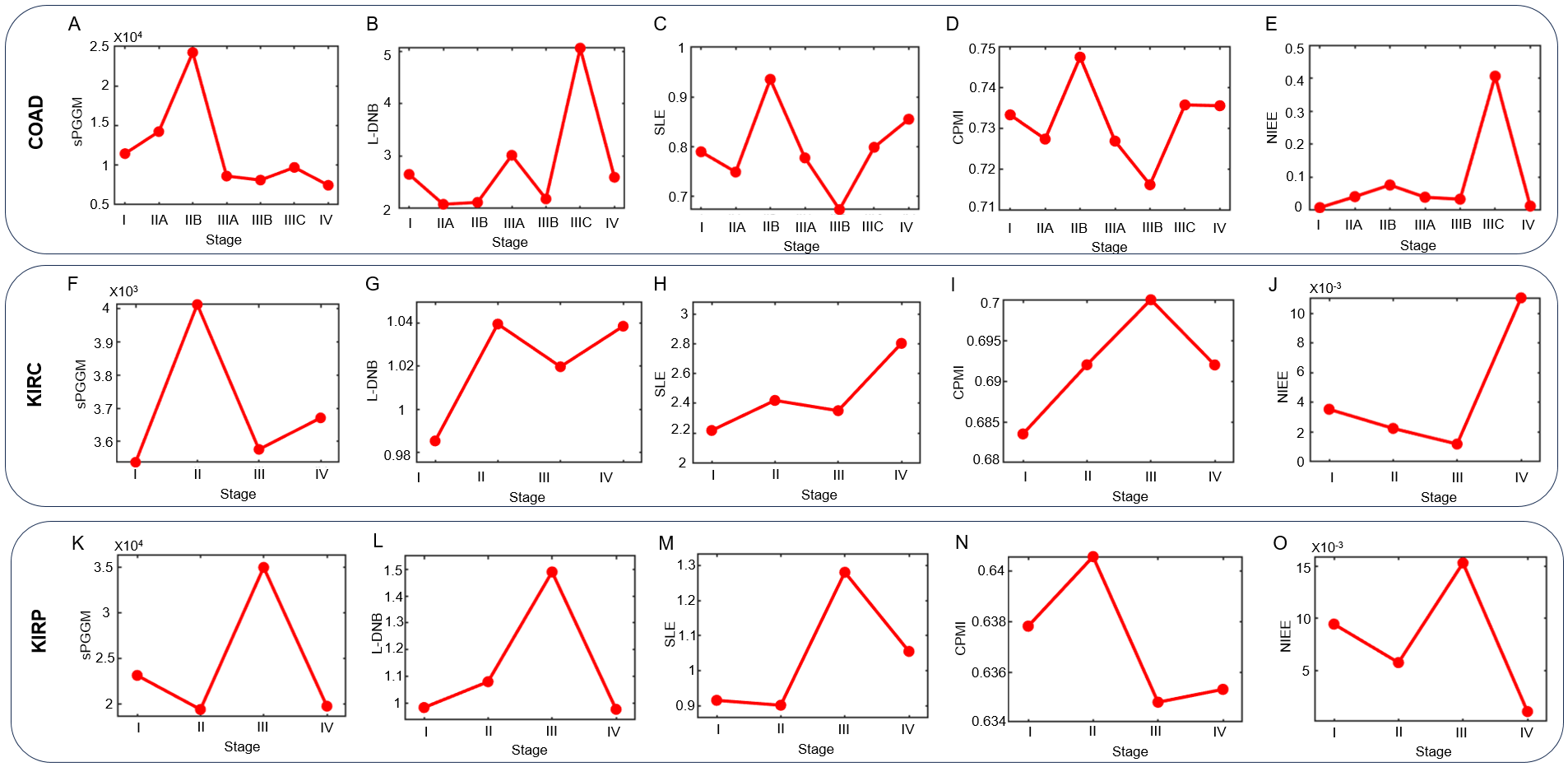


Figure S2. Comparison of the dynamic performance of various single-sample detection methods, including sPGGM, LDNB, SLE, CPMI, and NIEE, across three tumor datasets: (A)-(E) COAD, (F)-(J) KIRC, and (K)-(O) KIRP.


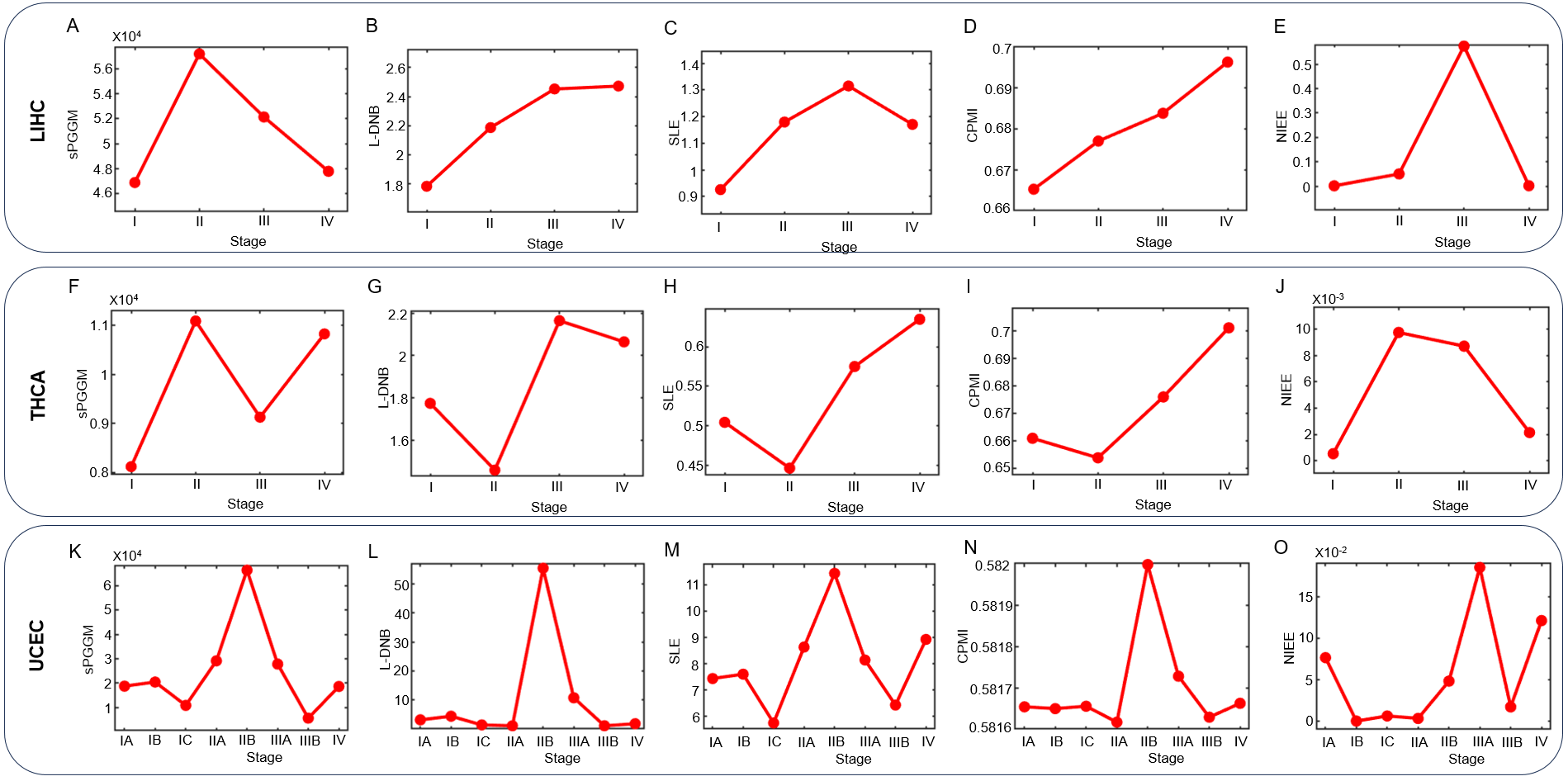


Figure S3. Comparison of the dynamic performance of various single-sample detection methods, including sPGGM, LDNB, SLE, CPMI, and NIEE, across three tumor datasets: (A)-(E) LIHC, (F)-(J) THCA, and (K)-(O) UCEC.


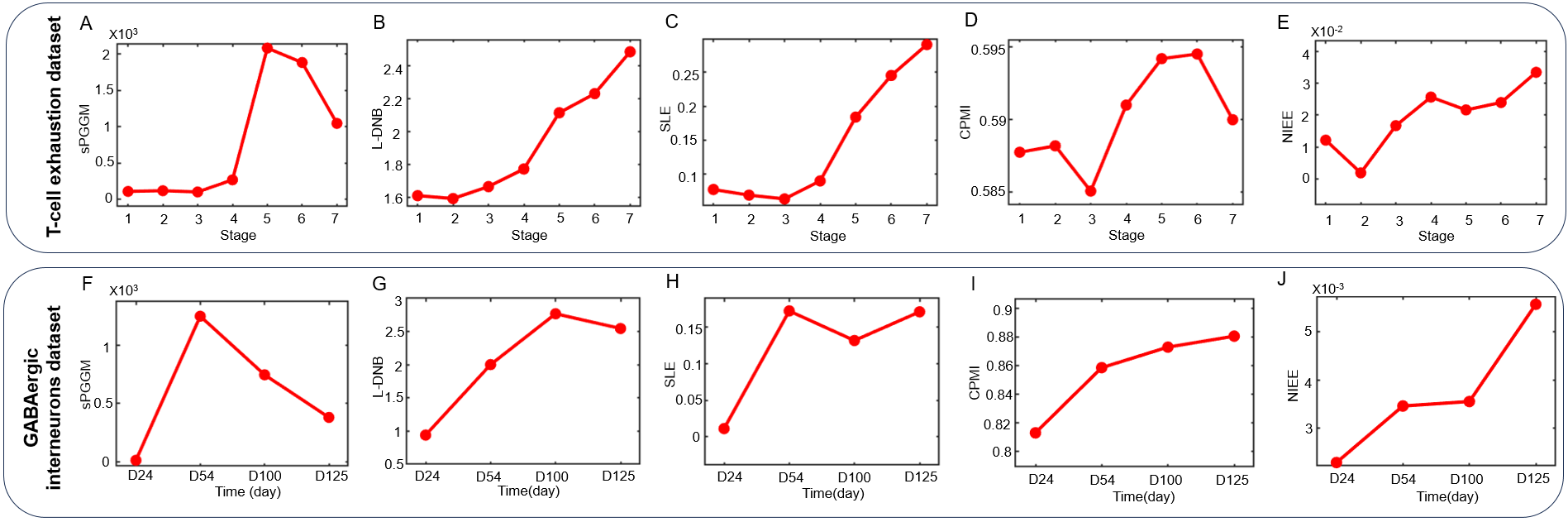


Figure S4. Assessment of the dynamic performance of various single-sample detection methods, including sPGGM, LDNB, SLE, CPMI, and NIEE, across two single-cell data: (A)-(E) T-cell exhaustion dataset, (F)-(J) GABAergic interneurons dataset.

**D.** **Description of stage transport map for the distribution of signaling molecules**

The transport map derived from the normal to abnormal state via sPGGM enables us to describe the disease progression using Gaussian graphical distributions. At the identified pre-disease stage, a group of genes called signaling molecules (the top 5% of genes exhibiting the highest sPGGM scores), was selected to gain insights into their role in disease development. Specifically，in this study, we utilize principal component analysis (PCA) [9] to demonstrate the main transformation processes in the distribution of signaling molecules at different stages of the disease. As shown in Figures S5A-C, the distribution transport process for three tumor datasets (LIHC, COAD, and UCEC) indicates that the distribution becomes increasingly concentrated as it moves away from critical points and gradually disperses as it approaches them. These observations not only emphasize the instability of the critical state and the sudden nature of deterioration, but also suggest that sPGGM effectively captures these distributional changes and identifies critical transitions across different diseases.


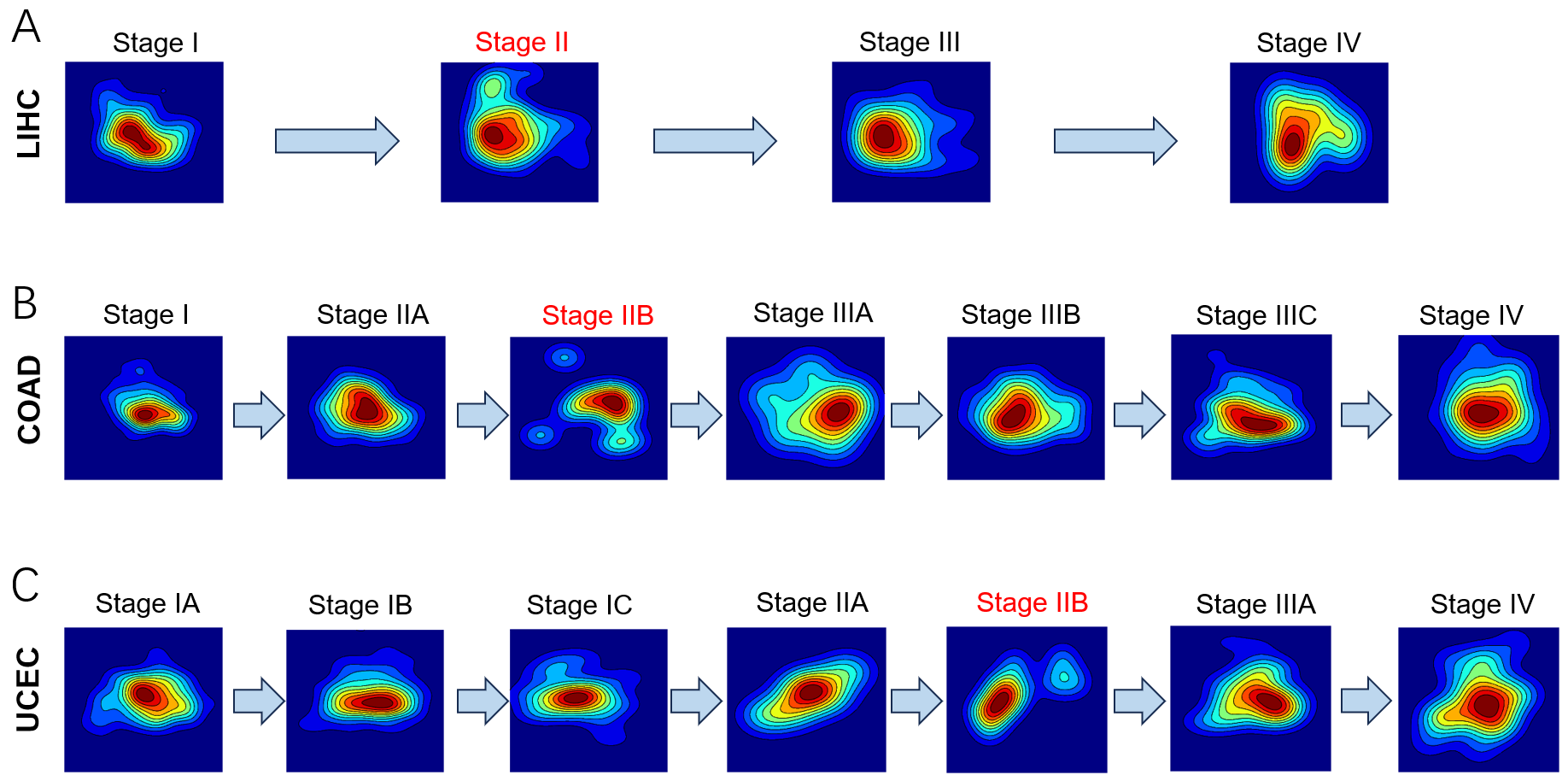


Figure S5. PCA-based visualizations of the optimal transport map transitioning from normal to abnormal states for three tumor types: (A) LIHC, (B) COAD, and (C) UCEC.

**E. Functional analysis of both dark molecule and DEG among** **signaling molecule**

In addition to the functional enrichment analysis of signaling molecules, we have also conducted an additional functional analysis for both "dark molecules" and differentially expressed genes (DEGs) among signaling molecule. As demonstrated in Figure S6A-B, both "dark molecules" and DEGs are significantly enriched in cancer-related pathways, with some shared signaling pathways such as Thermogenesis, Oxidative phosphorylation, and Chemical carcinogenesis - reactive oxygen species. For these common KEGG pathways, we observed that "dark molecules" tend to act as upstream regulators in the pathway, while DEGs are positioned as downstream effectors (Figure S6C), indicating that "dark genes" and DEGs function together in the process of cancer initiation and progression. This finding supports the idea that DEGs, in conjunction with "dark genes," may play a crucial role in the critical transition state in cancer.


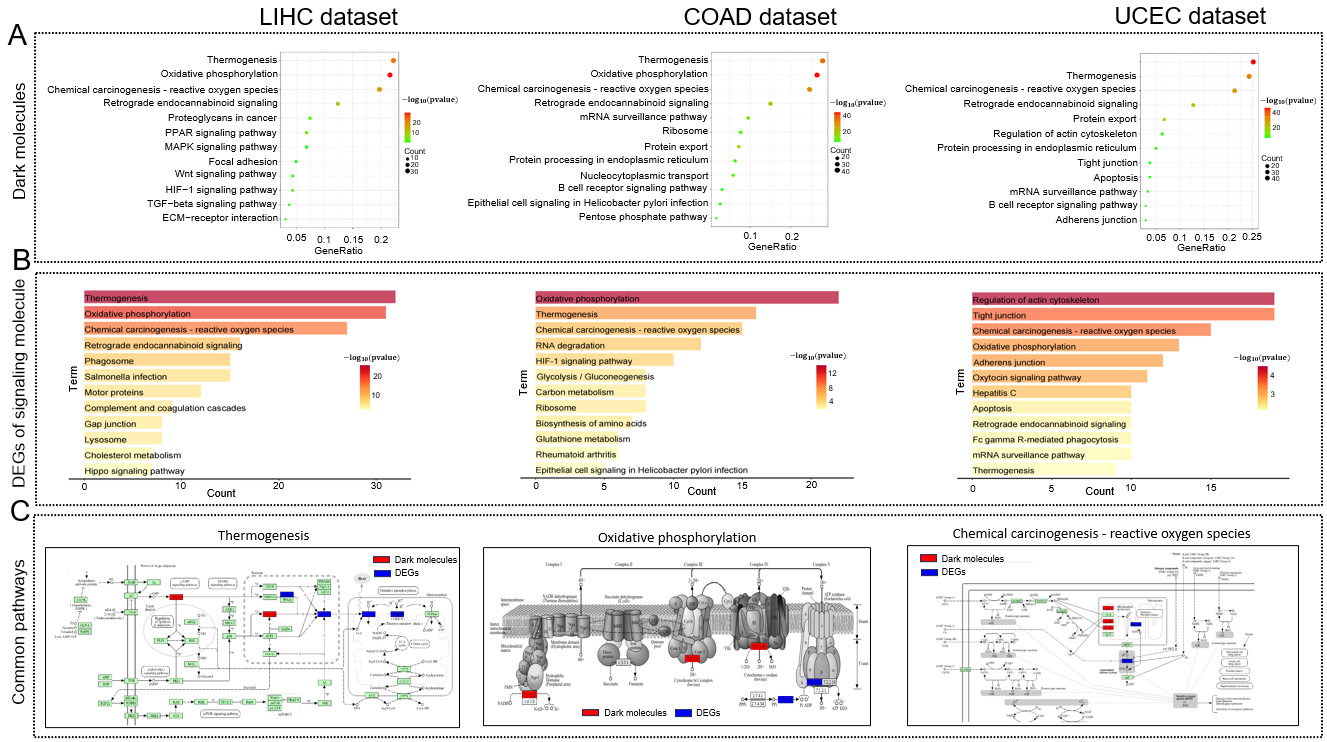


Figure S6. (A) KEGG pathway enrichment analysis of "dark molecules" in the three tumor types: LIHC, COAD, and UCEC. (B) KEGG pathway enrichment analysis of differentially expressed genes (DEGs) among signaling molecule in these three tumor types: LIHC, COAD, and UCEC. (C) For the common KEGG pathways, we observed that "dark molecules" primarily function as upstream regulators, while DEGs act as downstream effectors.

In most pathological and biomedical studies, differentially expressed genes are often the focus for identifying new biomarkers, key regulators, and drug targets. In our research, we define genes as "dark molecules" when they are non-differentially expressed yet sensitive to the sPGGM score. We discovered that certain "dark molecule" involved in cancer-related pathways are essential for disease progression and serve as effective prognostic indicators, not at the gene expression level but at the sPGGM score level (Figs. 5g-I of main text). These “dark molecules” sensitive to sPGGM score may play significant roles in key biological processes that trigger critical deterioration, but they are often overlooked due to their non-differential expression characteristics. Consequently, our sPGGM-based method can be viewed as a valuable complement to traditional differential expression analysis, helping to identify new biomarkers, drug targets, and prognostic indicators from a network-level perspective (since the sPGGM score is derived from network-based computations) rather than focusing solely on the gene level. To enhance the validation of "dark molecules" as prognostic indicators, we have conducted a comparison of survival analysis among the "dark molecules", the top 5% most significant DEGs, and randomly selected genes of the same size, using both sPGGM scores and gene expression values. For the LIHC (Figure S7 and Table S2) and COAD dataset (Figure S8 and Table S3), the "dark molecules" with the most significant prognostic p-values at the sPGGM score level and the DEGs with the most significant prognostic p-values at the expression level demonstrate that the sPGGM scores of the "dark molecules" perform as well as the expression levels of the DEGs in prognosis. In contrast, randomly selected genes showed no significant prognostic performance at either the sPGGM score or expression level. These results demonstrate that "dark molecules" are effective prognostic indicators at the sPGGM score level, while DEGs are effective at the expression level.


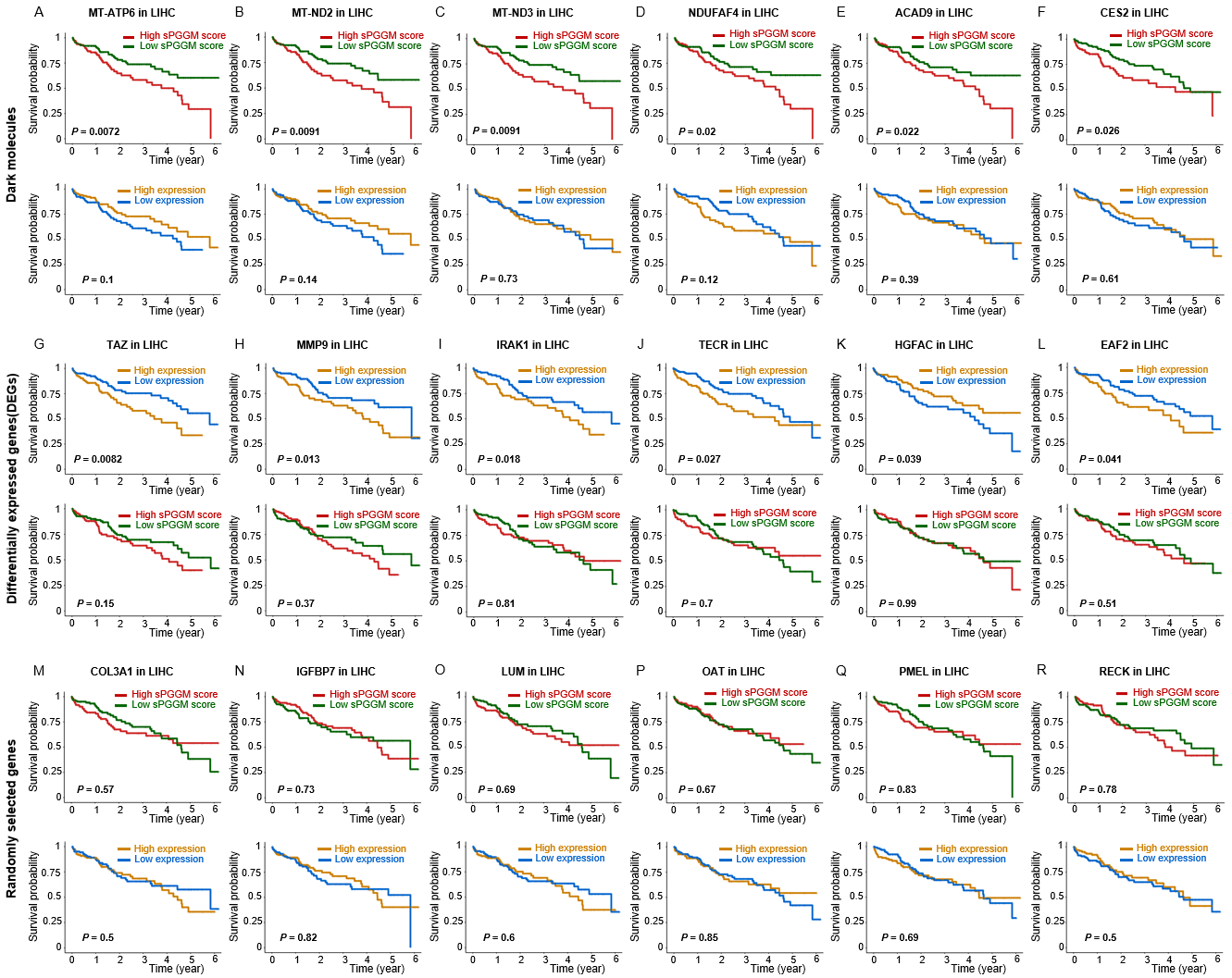


Figure S7. For LIHC dataset, a comparison of survival analysis among "dark molecules", the top 5% most significant DEGs, and randomly selected genes of the same size, based on both sPGGM scores and gene expression values. (A)-(F) Survival prognosis of the 'dark molecules' with the most significant p-values at the sPGGM score level. (G)-(L) Survival prognosis of the DEGs with the most significant p-values at the expression level. (M)-(R) Survival prognosis of the randomly selected genes.

**Table S2. The most significant prognostic p-value of three types of gene in LIHC dataset**

| **Dark molecules** | ***MT-ATP6*** | ***MT-ND2*** | ***MT-ND3*** | ***NDUFAF4*** | ***ACAD9*** | ***CES2*** |
| --- | --- | --- | --- | --- | --- | --- |
| **sPGGM level** | *P* = 0.0072 | *P* = 0.0091 | *P* = 0.0091 | *P* = 0.02 | *P* = 0.022 | *P* = 0.026 |
| **Expression level** | *P* = 0.1 | *P* = 0.14 | *P* = 0.73 | *P* = 0.12 | *P* = 0.39 | *P* = 0.61 |
| **DEGs** | ***TAZ*** | ***MMP9*** | ***IRAK1*** | ***TECR*** | ***HGFAC*** | ***EAF2*** |
| **Expression level** | *P* = 0.0082 | *P* = 0.013 | *P* = 0.018 | *P* = 0.027 | *P* = 0.039 | *P* = 0.041 |
| **sPGGM level** | *P* = 0.15 | *P* = 0.37 | *P* = 0.81 | *P* = 0.7 | *P* = 0.99 | *P* = 0.51 |
| **Randomly selected genes** | ***COL3A1*** | ***IGFBP7*** | ***LUM*** | ***OAT*** | ***PMEL*** | ***RECK*** |
| **sPGGM level** | *P* = 0.57 | *P* = 0.73 | *P* = 0.69 | *P* = 0.67 | *P* = 0.83 | *P* = 0.78 |
| **Expression level** | *P* = 0.5 | *P* = 0.82 | *P* = 0.6 | *P* = 0.85 | *P* = 0.69 | *P* = 0.5 |


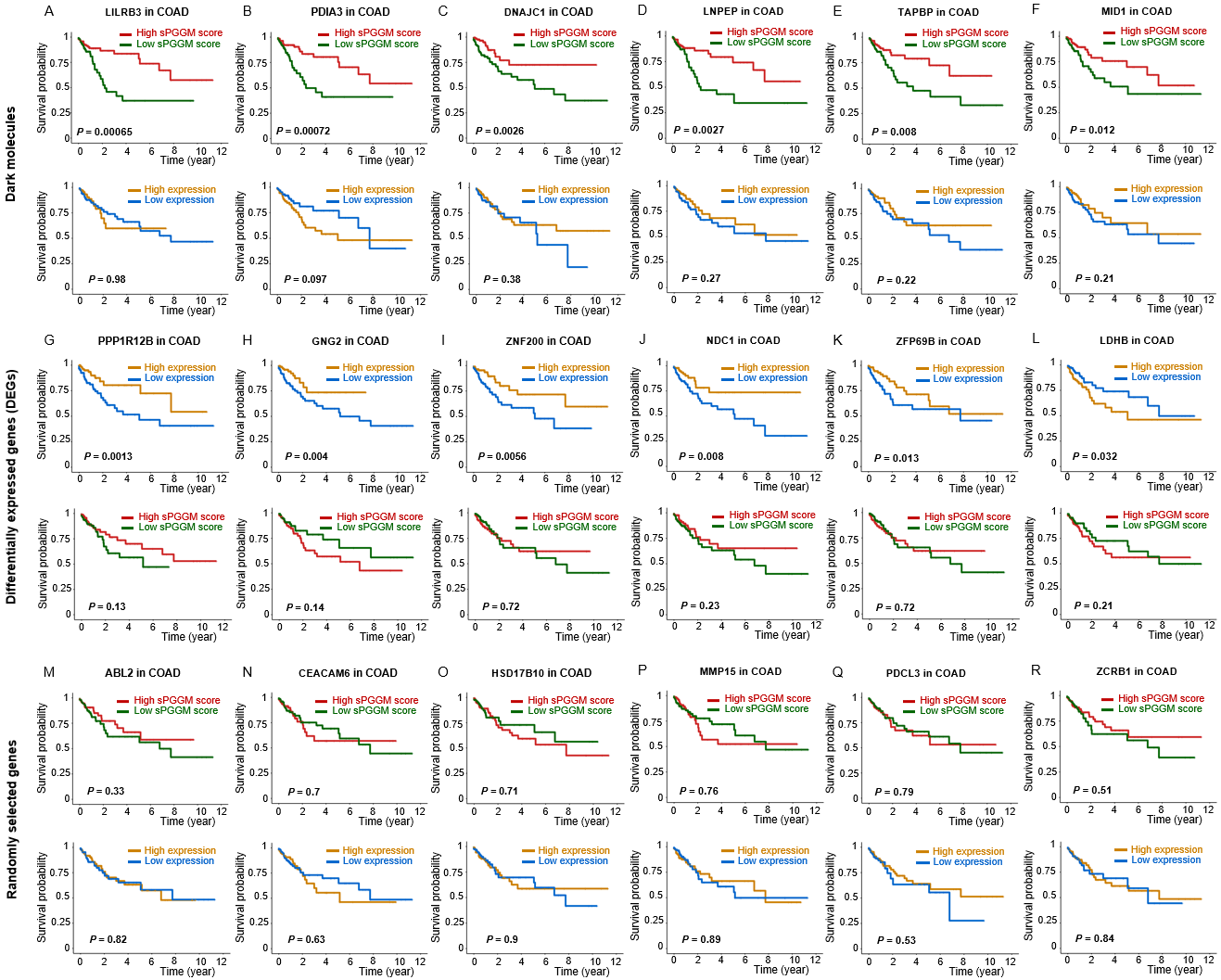


Figure S8. For COAD dataset, a comparison of survival analysis among "dark molecules", the top 5% most significant DEGs, and randomly selected genes of the same size, based on both sPGGM scores and gene expression values. (A)-(F) Survival prognosis of the 'dark molecules' with the most significant p-values at the sPGGM score level. (G)-(L) Survival prognosis of the DEGs with the most significant p-values at the expression level. (M)-(R) Survival prognosis of the randomly selected genes.

**Table S3. The most significant prognostic p-value of three types of gene in COAD dataset**

| **Dark molecules** | ***LILRB3*** | ***PDIA3*** | ***DNAJC1*** | ***LNPEP*** | ***TAPBP*** | ***MID1*** |
| --- | --- | --- | --- | --- | --- | --- |
| **sPGGM level** | *P* = 0.00065 | *P* = 0.00072 | *P =* 0.0026 | *P =* 0.0027 | *P =* 0.008 | *P* = 0.012 |
| **Expression level** | *P* = 0.98 | *P* = 0.097 | *P* = 0.38 | *P* = 0.27 | *P* = 0.22 | *P* = 0.21 |
| **DEGs** | ***PPP1R12B*** | ***GNG2*** | ***ZNF200*** | ***NDC1*** | ***ZFP69B*** | ***LDHB*** |
| **Expression level** | *P* = 0.0013 | *P* = 0.004 | *P* = 0.0056 | *P* = 0.008 | *P* = 0.013 | *P* = 0.032 |
| **sPGGM level** | *P* = 0.13 | *P* = 0.14 | *P =* 0.72 | *P* = 0.23 | *P* = 0.72 | *P* = 0.21 |
| **Randomly selected genes** | ***ABL2*** | ***CEACAM6*** | ***HSD17B10*** | ***MMP15*** | ***PDCL3*** | ***ZCRB1*** |
| **sPGGM level** | *P* = 0.33 | *P* = 0.7 | *P =* 0.71 | *P* = 0.76 | *P* = 0.79 | *P* = 0.51 |
| **Expression level** | *P* = 0.82 | *P* = 0.63 | *P* = 0.9 | *P* = 0.89 | *P* = 0.53 | *P* = 0.84 |

**F.** **Performance comparison of sPGGM and transition characterization methods**

We compare the performance of sPGGM with existing computational methods for characterizing transitions (including MuTrans [10], QuanTC [11], and BioTIP [12]) in the CD8+ T-cell exhaustion dataset and the GABAergic interneurons dataset. Specifically, MuTrans [10] and QuanTC [11] are primarily designed to dissect transition cells from scRNA-seq datasets. To quantify and dissect transition cells around different attractors from a global perspective, a transition entropy $H\left( x \right)$ for each cell $x$ is defined in literatures [10, 11] or as following Eq. S (5):

$H\left( x \right)=-\sum_{k=1}^{K} p_{xk}\log p_{xk},$ (S5)

where $p_{xk}$ represents the probability of cell $x$ belonging to the $k$-th attractor or state. As described in literatures [10, 11], a stable cell tends to exhibit a relatively small entropy value, whereas a transition cell, characterized by multiple and more evenly distributed components in its probabilities across different attractors, tends to have a larger transition entropy. Consequently, a large entropy value indicates a cell with highly mixing identity, characteristic of transition cells in bifurcating attractors. Therefore, the increase of transition entropy value can be utilized as a way to mark cell-state bifurcations or critical states of complex biological processes. As illustrated in Figure S9A, for the T-cell exhaustion dataset, sPGGM, MuTrans, and BioTIP detect the critical transition with significant increases ($P=1.2E-129$ for sPGGM; $P=9.4E-23$ for MuTrans; $P=3.7E-10$ for BioTIP), with sPGGM providing an earlier warning signal. When applied to the GABAergic interneurons dataset, it is seen from Figure S9B that sPGGM, MuTrans, QuanTC, and BioTIP consistently indicate the critical state at Day 54 ($P =0.018$ for sPGGM; $P =0.04$ for MuTrans; $P = 0.13$ for QuanTC; $P =0.058$ for BioTIP), with sPGGM showing the most statistically significant signal. Moreover, sPGGM is a sample-perturbed critical point detection model, meaning that it can detect critical signals at the specific sample/cell level when given a set of reference samples. Therefore, sPGGM has its own specific advantages in analyzing critical states.


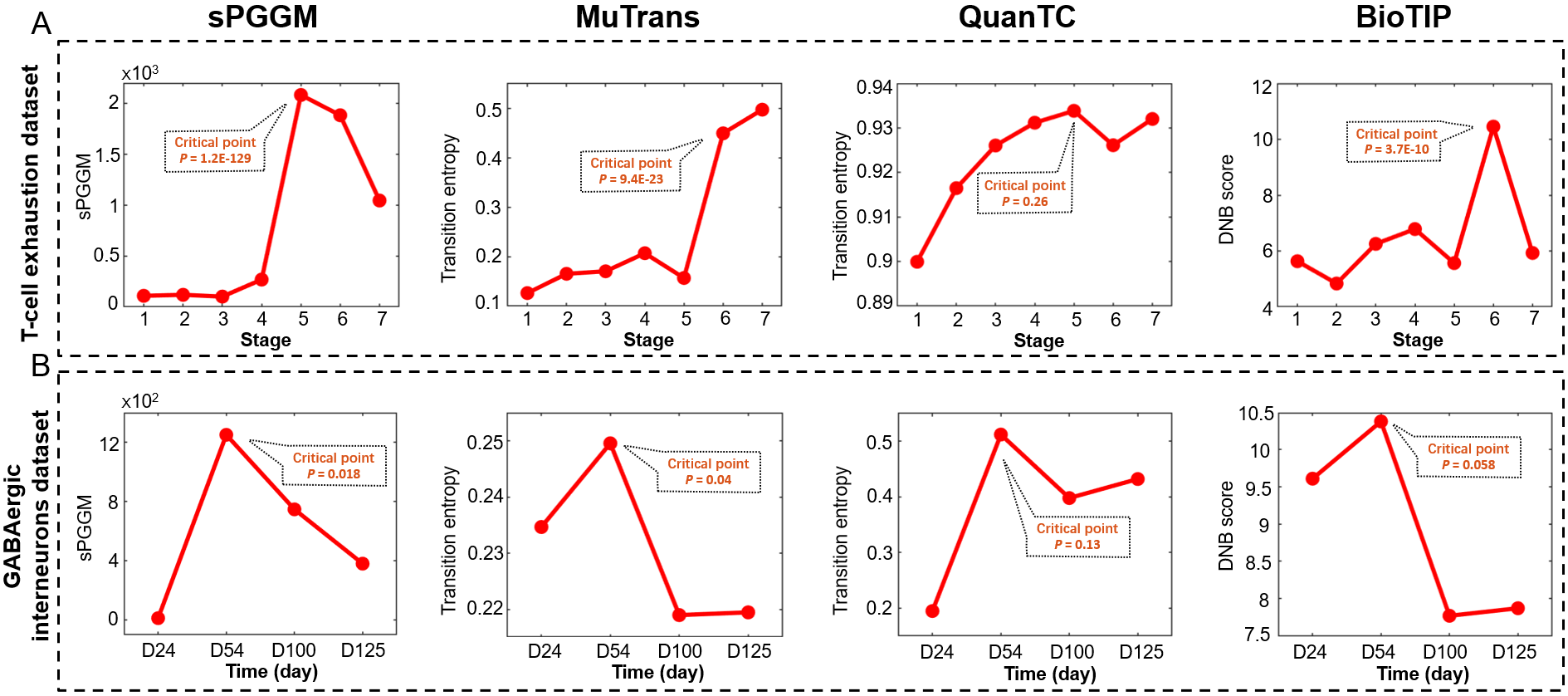


Figure S9. The dynamic performance of different transition characterization methods including sPGGM, MuTrans, QuanTC, and BioTIP was evaluated on the following single-cell datasets: (A) CD8+ T-cell exhaustion dataset and (B) GABAergic interneurons dataset.

**G. Functional analysis of the CD8+ T-cell exhaustion data**

Transcription factors (TFs) are known to be vital in regulating CD8+ T-cell exhaustion in colorectal cancer (CRC) by modulating the expression of associated target genes [13]. To examine the mechanism of signaling molecules, we analyzed the functions of those regulated by upstream transcription factors. Figure S10 revealed that 67% of the signaling molecules (dep orange-red circle) were regulated by TFs (red square), indicating their potential involvement in mediating CD8+ T-cell exhaustion in CRC.


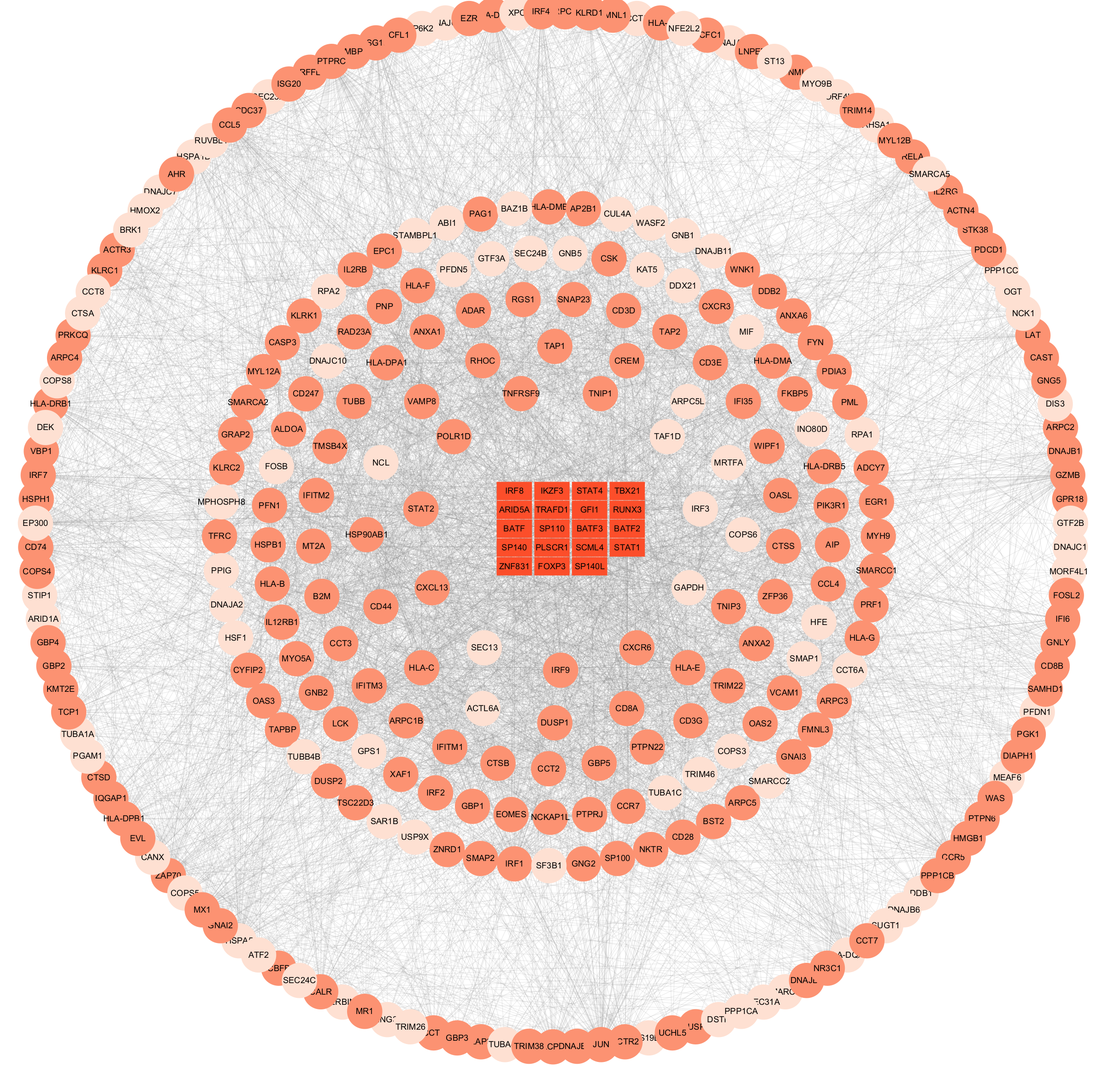


Figure S10. 67% of signaling molecules (dep orange-red circle) were regulated by transcription factors (red square).

It is observed that the upregulation of *RELA*, acting as a targeted signaling molecules regulated by transcription factors (TFs), drives the high expression of *PDCD1* and *PTPN6/PTPN11*, potentially playing a critical role in CD8+ T-cell exhaustion. *RELA, PDCD1, BATF, and PTPN6/11* exhibit significant expression differences before and after the critical point of CD8+ T-cell exhaustion (Figure S11), with markedly high levels in terminally exhausted CD8+ T-cell, indicating that these factors may serve as potential therapeutic targets to prevent CD8+ T-cell exhaustion.


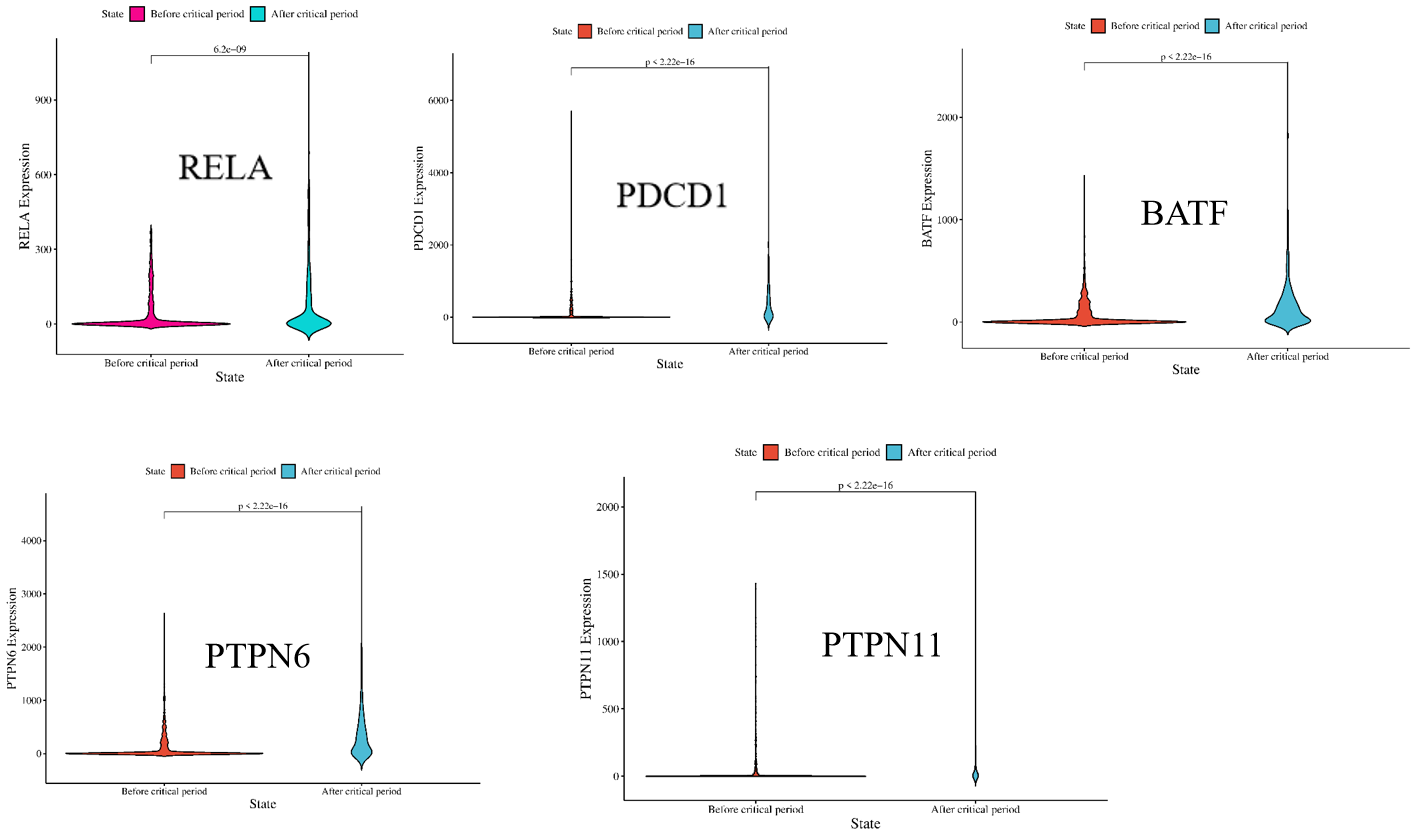


Figure S11. *RELA, PDCD1, BATF, and PTPN6/11* exhibit significant expression differences before and after the critical period of CD8+ T-cell exhaustion.

**H.** **Performance of sPGGM across different reference sample size**

To demonstrates the stability and robustness of the proposed sPGGM under different size of the reference group, we have performed a thorough analysis using a numerical simulation based on Eq.(S1). As depicted in Figure S12, sPGGM index accurately indicates the early warning signals of critical transition when the number of reference samples (the reference size)$N$ varies. Although an increase in the reference sample size tends to weaken critical signals (as indicated by a reduction in the maximum PGGM score shown in Figure S12), the number of reference samples in practical clinical applications is generally not excessively large, typically below 500. Therefore, the number of reference samples within a certain range does barely affect the effectiveness of the sPGGM index in detecting the critical state. In addition, we assessed the robustness of the sPGGM method by randomly selecting reference samples from a real-world T-cell exhaustion dataset at varying proportions, ranging from 10% to 100%. As illustrated in Figure S13, sPGGM index reliably identifies the critical transition point, thereby demonstrating that our method remains stable and robustness to perturbations introduced by varying proportions of reference samples.


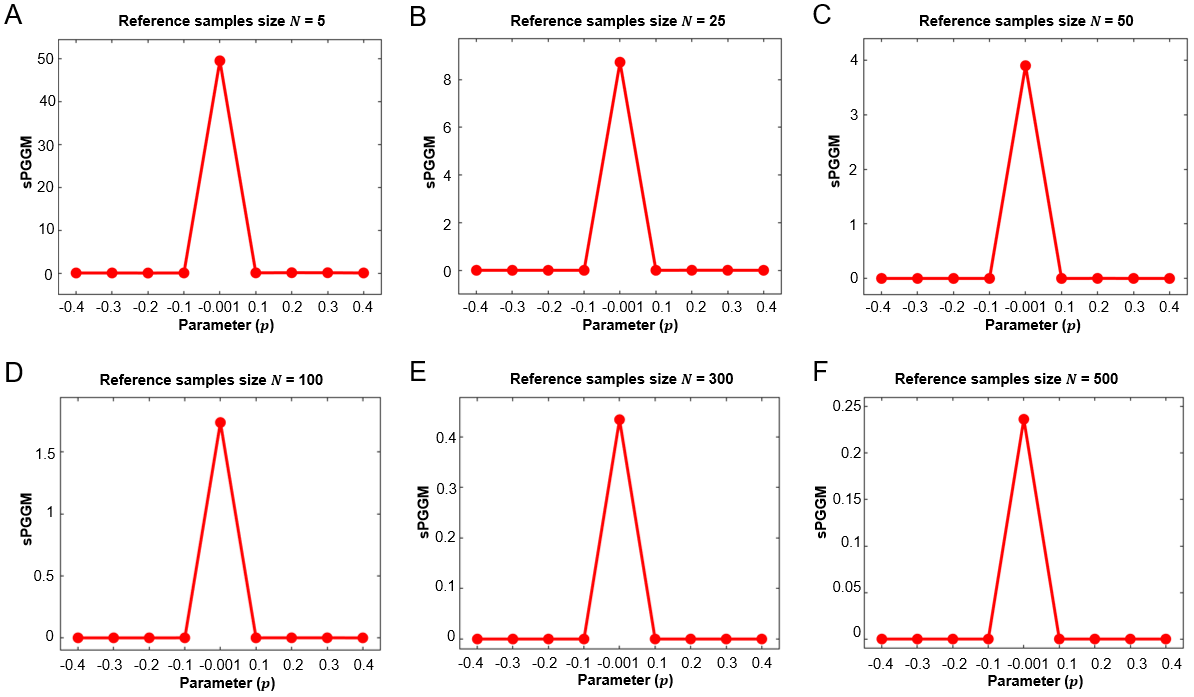


Figure S12. Performance of the sPGGM method with different reference sample sizes. Based on the numerical simulation dataset, we have analyzed the evolution tendency of the signal curve with different reference sample size $N$ for (A) sPGGM curve for reference sample size $N=5$, (B) sPGGM curve for reference sample size $N=25$, (C) sPGGM curve for reference sample size $N=50$, (D) sPGGM curve for reference sample size $N=100$, (E) sPGGM curve for reference sample size $N=300$, and (F) sPGGM curve for reference sample size $N=500$.

.


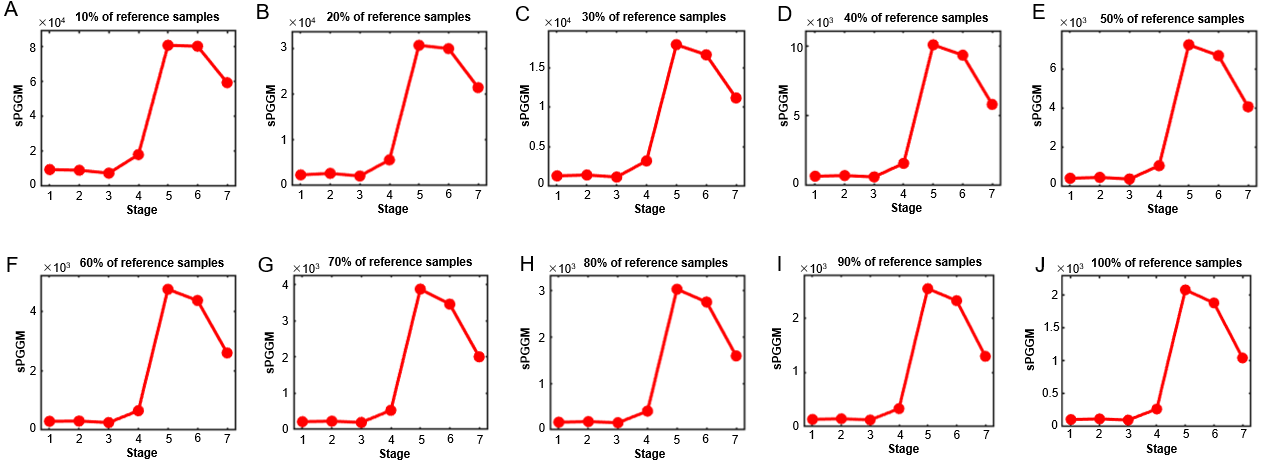


Figure S13. The robustness of the sPGGM method is evaluated by randomly selecting reference samples from a real-world T-cell exhaustion dataset at varying proportions ranging from 10% to 100%, as shown in panels (A) to (J). Therefore, the sPGGM index consistently identifies the critical transition point across different proportions of reference samples.

**I.** **Performance of sPGGM for analysing large single-cell datasets**

In addition to demonstrating the method's efficacy on the smaller datasets presented in the main manuscript, we have also applied sPGGM to two larger-scale datasets to rigorously assess its scalability and performance and also stated as follows. The first larger dataset [14] consists of around 2 million cells across five time points: E9.5, E10.5, E11.5, E12.5, and E13.5, which describe the process of mammalian organogenesis during embryonic development. It is seen from Figure S14A that the sPGGM score exhibits a rapid increase from E10.5 to E11.5 ($P =4.6E-9$), after which particularly dramatic changes occur, leading to the establishment of comprehensive organism genesis or mammalian organogenesis [14]. Another large dataset [15] comprises 356,213 cells from six age groups of C57BL/6JN mice, ranging from 1 month (equivalent to human infancy) to 30 months (comparable to human centenarians). This dataset provides an overview of cellular aging trajectories across these age groups. As shown in Figure S14B, a significant shift in the sPGGM score is observed at 18 months ($P = 4.7E-2$). This critical signal is consistent with the observations in the original literature that there is a marked increase in the expression of aging-related markers, reaching their peak at 24 and 31 months [15]. Above results demonstrate that sPGGM is scalable and effective for analyzing large single-cell datasets containing millions of cells, while also capturing biologically meaningful transitions.


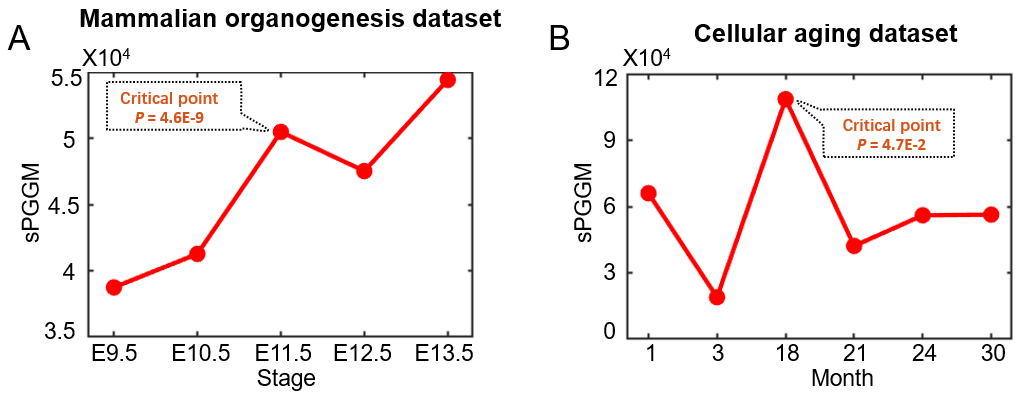


Figure S14. The dynamic behavior of sPGGM was evaluated across two larger-scale single-cell datasets: (A) mammalian organogenesis dataset and (B) cellular aging dataset. The results indicate that sPGGM can accurately detect upcoming critical transitions, demonstrating its scalability and effectiveness for analyzing large single-cell RNA-seq datasets.

**J.** **Derivation and proof of the 2-Wasserstein distance for Gaussian measures**

Let $U$ and $V$ be two probability measures in $\mathbb{R}^{d}$. Based on optimal transport theory [16], the 2-Wasserstein distance between $U$ and $V$ is defined as*:*

$W_{2}^{2}\left( U,V \right)=\inf_{T\#U=V} \int_{\mathbb{R}^{d}} \left\| x-T\left( x \right) \right\|_{2}^{2}dU\left( x \right),$ (S6)

where $T$ is the optimal transport map defined in $\mathbb{R}^{d}$ that minimizes the "transportation loss". The notation $T\#U$ represents the pushforward operator induced by the map $T,$ which is given by:

$T\#U(E)=U\left( \left\{ x\mid T\left( x \right)\in E \right\} \right)=U\left( T^{-1}\left( E \right) \right),$ (S7)

where $E\subset\mathbb{R}^{d}$ is arbitrary measurable subset. In addition, Kantorovich proposed an alternative formulation of the optimal transport problem [16], and as followed:

$W_{2}^{2}\left( U,V \right)=\inf_{\gamma\in J\left( U,V \right)} \int_{\mathbb{R}^{d}\times\mathbb{R}^{d}} \left\| x-y \right\|_{2}^{2}d\gamma\left( x,y \right)=\inf_{\gamma\in J\left( U,V \right)} E\left( \left\| X-Y \right\|_{2}^{2} \right),$ (S8)

Here, $J\left( U,V \right)$ denotes the set of joint distributions with marginals *U* and *V*，and *X* and *Y* are random variables distributed according to *U* and *V*, respectively. The optimal transport way $\gamma$ minimizes the loss function. Equations (S6) and (S8) are equivalent under certain conditions, but in general, they are challenging to solve.

Assuming the random vectors $X$ and *Y* follow two $d$-dimensional Gaussian distributions $U=N\left( \mu_{U},\Sigma_{U} \right)$ and $, V=N(\mu_{V},\Sigma_{V}),$ we can derive the following equation when $\xi=X-\mu_{U}$ and $\eta=Y-\mu_{V}$:

$E\left\| X-Y \right\|_{2}^{2}=\left\| \mu_{U}-\mu_{V} \right\|_{2}^{2}+E\left\| \xi-\eta\right\|_{2}^{2}.$ (S9)

Then the 2-Wasserstein distance between $U$ and $V$ can be expressed as:

$W_{2}^{2}\left( U,V \right)=\left\| \mu_{U}-\mu_{V} \right\|_{2}^{2}+W_{2}^{2}\left( P_{U},P_{V} \right),$ (S10)

where $P_{U}=N\left( 0,\Sigma_{U} \right) \mathrm{and} P_{V}=N(0,\Sigma_{V})$. Therefore, we focus on the 2-Wasserstein distance between the two zero-mean Gaussians $P_{U}$ and $P_{V}$. The optimal transport way between $P_{U}$ and $P_{V}$ can be written as a $2d$-dimensional Gaussian distribution $N(0,A)$, where $A$ is a block matrix given by [17]：

$A=\left( \begin{matrix} \Sigma_{U} & K \\ K^{T} & \Sigma_{V} \end{matrix} \right).$ (S11)

Here, *K* is a $d\times d$ matrix. Consequently, we have:

$E\left\| \xi-\eta\right\|_{2}^{2}=E\left( \xi^{T}\xi\right)+E\left( \eta^{T}\eta\right)-2E\left( \xi^{T}\eta\right)=\mathrm{trace} \Sigma_{U}+\mathrm{trace} \Sigma_{V}-2\mathrm{trace} K.$ (S12)

Now, the problem reduces to finding a matrix *K* that minimizes equation (S12), while ensuring that *A* remains positive semidefinite. The covariance matrix *A* can be factorized as:

$A=\left( \begin{matrix} \Sigma_{U}^{1/2} & 0 \\ K^{T}\Sigma_{U}^{-1/2} & I \end{matrix} \right)\left( \begin{matrix} I & 0 \\ 0 & S \end{matrix} \right)\left( \begin{matrix} \Sigma_{U}^{1/2} & \Sigma_{U}^{-1/2}K \\ 0 & I \end{matrix} \right)$ , (S13)

where $S=\Sigma_{V}-K^{T}\Sigma_{U}^{-1}K$ is the Schur complement. Since both $\Sigma_{U} \mathrm{and} \Sigma_{V}$ are positive definite matrices，their square roots $\Sigma_{U}^{1/2} \mathrm{and} \Sigma_{V}^{1/2}$, as well as their inverses, are also positive definite matrices. From the factorization of *A*，it follows that *A* is a positive semidefinite $2d$-dimensional matrix if $S$ is a positive semidefinite$d$-dimensional matrix. As $\Sigma_{U}^{-1}$ is positive definite, $\Sigma_{V}-S=K^{T}\Sigma_{U}^{-1}K$ is also positive semidefinite. If the rank of $\Sigma_{V}-S$ is $r$, we have the spectral decomposition:

$\Sigma_{V}-S=Q\Lambda^{2}Q^{T}=Q_{r}\Lambda_{r}^{2}Q_{r}^{T},$ (S14)

where $\Lambda^{2}=\mathrm{diag} \left( \lambda_{1}^{2},\ldots,\lambda_{r}^{2},0,\ldots,0 \right)=\Lambda_{r}^{2}\bigoplus0$ is a diagonal matrix with $r$ nonzero eigenvalues, and $Q=[Q_{r},Q_{d-r}]$ is a matrix of corresponding orthonormal eigenvectors. The$\Lambda_{r}=\mathrm{diag} \left( \pm\lambda_{1},\ldots,\pm\lambda_{r} \right)$ is an invertible diagonal matrix. Let $O=\Sigma_{U}^{-1/2}{KQ}_{r}\Lambda_{r}^{-1}$，and from equation (S14), we deduce that:

$O^{T}O=\Lambda_{r}^{-1}Q_{r}^{T}\left( \Sigma_{V}-S \right)Q_{r}\Lambda_{r}^{-1}=\Lambda_{r}^{-1}\Lambda_{r}^{2}\Lambda_{r}^{-1}=I_{r},$ (S15)

where $I_{r}$ is the identity matrix of size $r$. Thus, $KQ_{r}=\Sigma_{U}^{1/2}O\Lambda_{r}$. Since $\Sigma_{U}^{-1}$ is positive definite, it follows that $KQ_{d-r}=0$. Then we have:

$K=KQQ^{T}=KQ_{r}Q_{r}^{T}=\Sigma_{U}^{1/2}O\Lambda_{r}Q_{r}^{T}$ *.* (S16)

The problem now can be expressed by minimizing the following:

$f\left( O \right)=\mathrm{trace} \Sigma_{U}+\mathrm{trace} \Sigma_{V}-2\mathrm{trace} \left( O^{T}B \right),$ (S17)

where $O$ and $B=\Sigma_{U}^{1/2}Q_{r}\Lambda_{r}$ are $d\times r$ matrices satisfying $O^{T}O=I$, and *B* is full rank. Let $O=[v_{1},\ldots,v_{r}]$ represents the columns of $O$，we can apply the method of Lagrange multipliers to find the extremum. From $O^{T}O=I$, we obtain the condition $v_{i}^{T}v_{j}=\delta_{ij}$（where $\delta_{ij}=1$ if $i=j$, and $\delta_{ij}=0$, if $i\neq j$）, leading to the auxiliary function:

$\sum_{i,j} c_{ij}(v_{i}^{T}v_{j}-\delta_{ij}), c_{ij}=c_{ji}.$ (S18)

In matrix form, this can be written as $\mathrm{trace} \left[ C(O^{T}O-I) \right]$, where $C$ is a $r\times r$ symmetric matrix acting as the Lagrange multiplier. Consequently, the auxiliary function becomes:

$F\left( O,C \right)=\mathrm{trace} (\Sigma_{U}+\Sigma_{V})-2\mathrm{trace} \left( O^{T}B \right)+\mathrm{trace} \left[ C\left( O^{T}O-I \right) \right].$(S19)

By solving the conditions $F_{O}=F_{C}=0$，we obtain $OC=B$ and $O^{T}O=I$. Since both *O* and *B* have of full rank, $C^{-1}$ exists and $C=O^{T}B$. Substituting this result, we have:

$I_{r}=O^{T}O=\left( BC^{-1} \right)^{T}\left( BC^{-1} \right)=\left( C^{-1} \right)^{T}B^{T}BC^{-1}.$ (S20)

Furthermore, it follows that: $C^{2}=B^{T}B$. Therefore，$f=\mathrm{trace} \Sigma_{U}+\mathrm{trace} \Sigma_{V}-2\mathrm{trace} \left( O^{T}B \right)$ is minimized by taking the positive definite square root of $B^{T}B$. Substituting the result from the Lagrange multiplier method into equation (S17), where $B=\Sigma_{U}^{1/2}Q_{r}\Lambda_{r}$, we obtain

$$f_{\min}=\mathrm{trace} \Sigma_{U}+\mathrm{trace} \Sigma_{V}-2\mathrm{trace} \left( \sqrt{B^{T}B} \right)$$

$$=\mathrm{trace} \Sigma_{U}+\mathrm{trace} \Sigma_{V}-2\mathrm{trace} \left( \sqrt{BB^{T}} \right)$$

$$=\mathrm{trace} \Sigma_{U}+\mathrm{trace} \Sigma_{V}-2\mathrm{trace}\left( \sqrt{\Sigma_{U}^{1/2}Q_{r}\Lambda_{r}^{2}Q_{r}^{T}\Sigma_{U}^{1/2}} \right)$$

$=\mathrm{trace} \Sigma_{U}+\mathrm{trace} \Sigma_{V}-2\mathrm{trace} \left( \sqrt{\Sigma_{U}^{1/2}(\Sigma_{V}-S)\Sigma_{U}^{1/2}} \right).$ (S21)

Let $\mu_{1}^{2}\left( S \right)\geq\cdots\geq\mu_{d}^{2}(S)$ denote the eigenvalues of $\Sigma_{U}^{1/2}(\Sigma_{V}-S)\Sigma_{U}^{1/2}$. Note that *S* is positive semi-definite, so for any vector *v,* we have:

$v^{T}\Sigma_{U}^{1/2}\left( \Sigma_{V}^{1/2}-S \right)\Sigma_{U}^{1/2}v\leq v^{T}\Sigma_{U}^{1/2}\Sigma_{V}^{1/2}\Sigma_{U}^{1/2}v.$ (S22)

Because this inequality persists when *v* is constrained to various subspace of $\mathbb{R}^{d}$, we conclude from the Courant-Fischer theorem [17] that: $\mu_{j}^{2}\left( S \right)\leq\mu_{j}^{2}\left( 0 \right), j=1,\ldots,d$, and thus:

$f_{\min}=\mathrm{trace} \Sigma_{U}+\mathrm{trace} \Sigma_{V}-2\mathrm{trace} \left( \sqrt{\Sigma_{U}^{1/2}\Sigma_{V}\Sigma_{U}^{1/2}} \right)$ . (S23)

Combining this result with Equation (S10)，the 2-Wasserstein distance between $N\left( \mu_{U},\Sigma_{U} \right)$ and $N(\mu_{V},\Sigma_{V})$ is given by:

$W_{2}^{2}\left( U,V \right)=\left\| \mu_{U}-\mu_{V} \right\|_{2}^{2}+\mathrm{trace} \left( \Sigma_{U}+\Sigma_{V}-2\sqrt{\Sigma_{U}^{1/2}\Sigma_{V}\Sigma_{U}^{1/2}} \right).$(S24)

Additionally, the optimal transport map [18] can be derived as:

$T\left( x \right)=\mu_{V}+\Sigma_{U}^{-1/2}\left( \Sigma_{U}^{1/2}\Sigma_{V}\Sigma_{U}^{1/2} \right)^{1/2}\Sigma_{U}^{-1/2}(x-\mu_{U}).$ (S25)

**K.** **Performance of sPGGM at different PPI confidence thresholds**

To evaluate the effect of different confidence thresholds on the sPGGM index, we performed a comparative analysis using UCEC dataset to investigate the performance of sPGGM in detecting critical transitions at confidence thresholds of 0.9, 0.7, and 0.5. As shown in Figure S15, the signal strength provided by a confidence threshold of 0.9 is significantly stronger than that observed for thresholds of 0.7 and 0.5. Therefore, in this research, we utilized the graph G derived from a confidence threshold of 0.9, as it demonstrated improved performance in capturing the dynamic changes of complex diseases progression. Additionally, we also explored how the degree of local graphs influences the fitted Gaussian distribution. When applied to UCEC dataset and simulated data, as illustrated in Figure S16, as the local graph's number of nodes (i.e., degree) increases, the difference (quantified by the Local sPGGM score as outlined in Eq. (6) of the main text) between its baseline distribution (fitted from reference samples) and perturbed distribution (fitted from mixed samples that combine a specific case sample with the reference group) decreases at the critical point.


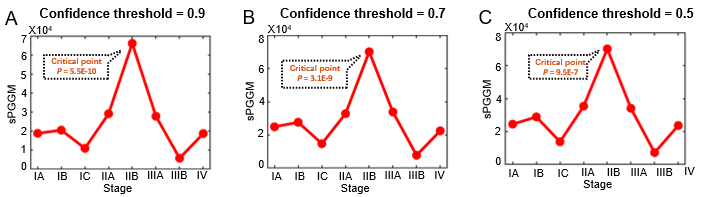


Figure S15. Comparison of the dynamic performance of sPGGM at different confidence thresholds. We analyzed the signal strength of the critical state for (A) a confidence threshold of 0.9, (B) a confidence threshold of 0.7, and (C) a confidence threshold of 0.5. The signal strength provided by a confidence threshold of 0.9 is stronger than that observed for thresholds of 0.7 and 0.5.


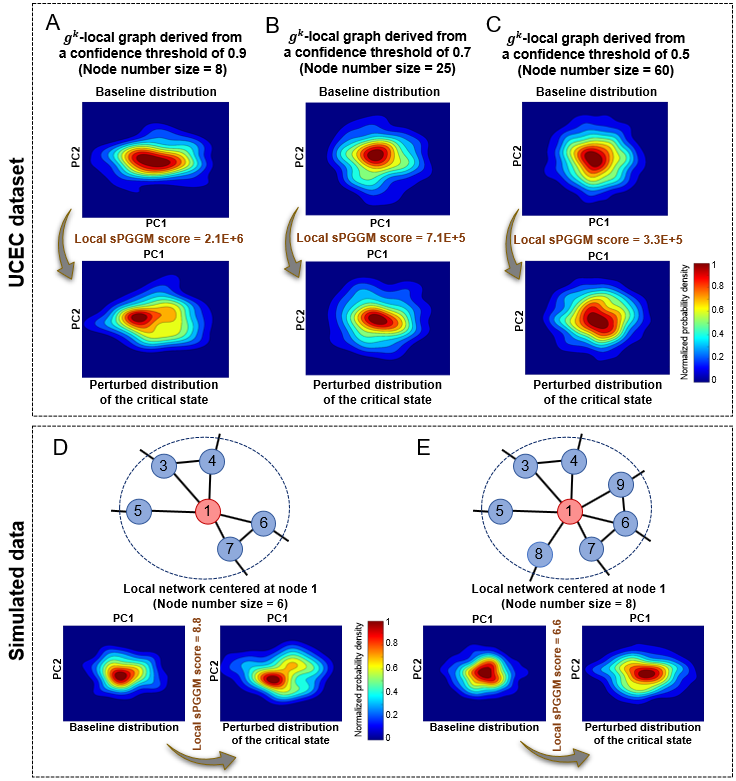


Figure S16. The relationship between the local graph's number of nodes (i.e., degree) and its fitted baseline distribution and perturbed distribution. The results are shown for (A)-(C) UCEC dataset and (D)-(E) simulated data. It was found that if the degree of the local graph increases, the difference between its baseline and perturbed distributions decreases at the critical point.

**L. Comprehensive explanation for focusing on 1st-order interactions**

In this study, each local graph can be derived from the global gene graph $G$. For a given gene $g^{k}$, the corresponding $g^{k}$-local graph ${LG}^{k}$ is centered around the gene itself, with its 1st-order neighbors $\left\{ g_{1}^{k}, g_{2}^{k},\ldots, g_{Q}^{k} \right\}$ constituting the $Q$ edges of the local graph. A comprehensive explanation for focusing on first-order interactions is stated as follows: (1) Priority of biological significance: First-order interactions are of paramount biological significance in gene graphs, often serving as the core drivers of higher-order interactions (e.g., second-order, third-order, etc.). Specifically, higher-order interactions frequently build upon the foundational first-order relationships. (2) Representation of essential information: First-order interactions generally provide the essential information for capturing the core structural and functional patterns of gene expression, while higher-order interactions contribute relatively little to the overall informational content. (3) Simplification of complexity: First-order interactions provide a more tractable and interpretable framework for uncovering the fundamental structure and functional principles of graphs.

Additionally, we performed a comparative analysis using COAD and UCEC dataset to evaluate two scenarios: one considering all genes within the global graph (without specific gene selection), and another focusing on cancer-related genes derived from the KEGG database. As shown in Figure S17, the signal strength of the model incorporating all genes is significantly stronger than that of the cancer-related gene selection approach. As a data-driven model, sPGGM yields better results by considering all genes within the global networks, as it avoids overlooking dynamic network biomarkers (DNBs) that play a critical role in detecting early warning of critical transitions. Therefore, in this study, we utilized all genes within the global graph, which demonstrated improved performance in analyzing the dynamic changes of complex diseases progression.


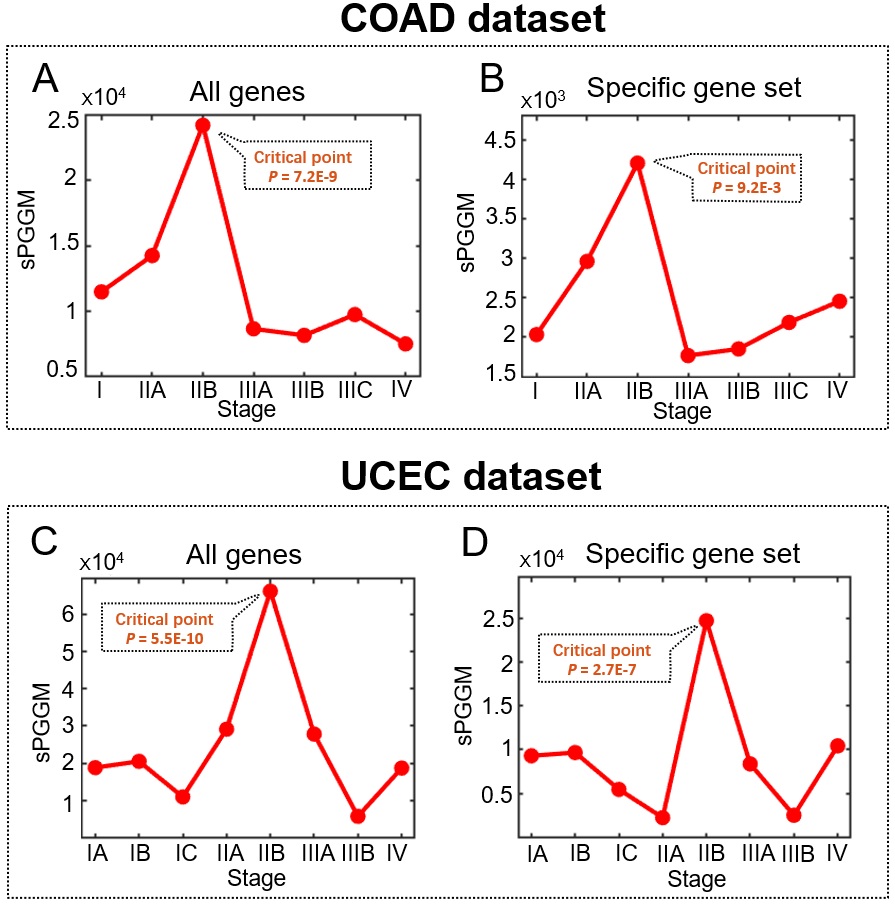


Figure S17. Comparison of dynamic changes performance of sPGGM across two scenarios: one considering all genes within the global graph (without specific gene selection), and another focusing on cancer-related genes derived from the KEGG database. The performance is assessed for (A)-(B) COAD dataset, and (C)-(D) UCEC dataset. The signal strength of the model incorporating all genes (P-value = 7.2E-9 for COAD, P-value =5.5E-10 for UCEC) is significantly stronger than that of the cancer-related gene selection (specific gene set) approach (P-value =9.2E-3 for COAD; P-value=2.7E-7 for UCEC).

**M.** **Description of unveiling the** **pre-disease stage**

To assess the effectiveness of the sPGGM score in capturing critical behavior, we utilize a one-sample test to evaluate whether a statistically significant difference exists between the normal and pre-disease stages. Specifically, the one-sample test statistic $ZS$ can be found below Eq. (S26) and is employed to assess whether the constant $y$ shows a significant deviation from the mean of the vector $\hat{Y}=(y_{1},y_{2},\cdots,y_{n})$:

$ZS= \frac{(\mathrm{mean}\left( \hat{Y} \right)-y)\sqrt{n}}{\mathrm{SD}\left( \hat{Y} \right)},$ (S26)

where the term $\mathrm{mean}\left( \hat{Y} \right)$ represents the average of vector $\hat{Y}$,and $\mathrm{SD}\left( Y \right)$ refers to its standard deviation. The P-value related to the index is used to evaluate the statistical difference between $\mathrm{mean}\left( \hat{Y} \right)$ and $y$. If the P-value is less than or equal to 0.05 ($P<0.05$), it indicates a significant statistical difference between $\mathrm{mean}\left( \hat{Y} \right)$ and $y$. In our research, a time point $t>2$ is classified as pre-disease stage or critical state if the sPGGM score $D_{t}$ meets the following two conditions: (i)$D_{t}>D_{t-1}$ and  (ii) $D_{t}$ shows a statistically significant difference ($P<0.05$) compared to the average of the vector $D$=$(D_{1},D_{2},\cdots,D_{t-1})$. When the following criteria is satisfied by the sPGGM score $D_{2}$: (i) $D_{2}$>$D_{1}$ and (ii) $D_{2}$ is statistically different ($P<0.05$) from the average of the vector $D$=$(D_{1},D_{3})$, the time point $t=2$ is viewed as the pre-disease stage.

**N. Materials and Methods**

**Theoretical background**

Based on our recently proposed theoretical concept of DNB [19, 20], as the system nears a critical point, a set of molecules (DNB variables) with strong correlations and large fluctuations emerges, signalling an impending critical state transition from a network-level perspective. It is evident that the state shift or phase of a system can be characterized by a dynamic change in both the multivariate distribution and molecular associations of DNB members. Therefore, by applying the Gaussian graphical model and optimal transport theory, we introduce sPGGM to detect critical signals marking the key transition from the normal stage to the disease stage, which identifies the pre-disease stage from a sample-specific perspective and addresses challenges such as small sample sizes, high noise levels, and sample heterogeneity. In our study, the Gaussian graphical model is defined by a graph structure paired with a Gaussian distribution, enabling the graph to depict the dependencies among molecules within the multivariate Gaussian distribution [21-23]. Specifically, the $d$-dimensional random vector $X\in\mathbb{R}^{d}$ is distributed according to a multivariate Gaussian distribution $N(\mu,\sum)$, where $\mu\in\mathbb{R}^{d}$ represents the mean vector, and $\sum\in\mathbb{R}^{d\times d}$ denotes the covariance matrix, which is symmetric and positive semi-definite. The corresponding probability density function of the multivariate Gaussian distribution can be expressed as:

$f\left( X \right)=\frac{1}{{(2\pi)}^{\frac{d}{2}}{(det(\sum))}^{\frac{1}{2}}}\exp\left\{ -\frac{1}{2}{(X-\mu)}^{T}\sum^{-1}\left( X-\mu\right) \right\}.$ (S27)

Given $n$ independent samples $X_{j}=\left( x_{1j},x_{2j},\ldots,x_{dj} \right)^{T}(j=1,\ldots,n)$, the log-likelihood function can be expressed as:

$\mathcal{l}\left( \mu,\Sigma\right)\propto-\frac{n}{2}\log\det\Sigma-\frac{1}{2}\sum_{j=1}^{n} \left( X_{j}-\mu\right)^{T}\Sigma^{-1}\left( X_{j}-\mu\right).$ (S28)

The unbiased estimate of $\mu$ and $\Sigma$ are derived by:

$\hat{\mu}=\frac{1}{n}\sum_{j=1}^{n} X_{j}, \hat{\Sigma}=\frac{1}{n-1}\sum_{j=1}^{n} \left( X_{j}-\hat{\mu} \right)\cdot\left( X_{j}-\hat{\mu} \right)^{T}.$ (S29)

Moreover, let $G=(V,E)$ represent a graph, where $V$ corresponds to the $d$ elements in $X$ and $E$ represents the edges connecting these elements, with each edge signifying a dependency between two elements. If the random vector $X$ follows a multivariate Gaussian distribution $N(\mu,\sum)$ such that $\sum^{-1}(i,j)$ =0 for every $(i,j)\notin E$, then it is considered to be governed by the Gaussian graphical model associated with the graph $G$[21, 23]. Clearly, graph $G$ can reflect the sparseness structure of the precision matrix $\sum^{-1}$, where the absence of edges represents conditional independence relationships in the Gaussian graphical model.

To explore the critical transition of the complex system, we introduce optimal transport theory for distribution changes, which offers a well-defined distance metric, namely the Wasserstein distance between distributions, and a geometry-based method to establish couplings between two probability distributions. Specially, let $U$ and $V$ be two measures in $\mathbb{R}^{d}$, the Wasserstein distance between two data distributions $U$ and $V$ is defined as follows:

$W_{2}^{2}\left( U,V \right)=\inf_{T_{\#}U=V} \int_{\mathbb{R}^{d}} \left| \left| x-T\left( x \right) \right| \right|_{2}^{2}dU\left( x \right),$ (S30)

where $T$ is denote the optimal transport map that corresponds to the smallest “cost” associated with the distance between $x$ and $T\left( x \right)$ in the space $\mathbb{R}^{d}$, and $T_{\#}U$ refers to the push-forward operation from $U$ to $V$ i.e. the equation $U\left( T^{-1}\left( A \right) \right)=V(A)$ holds for all measurable subset $A$ in $\mathbb{R}^{d}$. It is difficult to solve the formulation (S30). However, when $U$ and $V$ are multivariate Gaussian distribution with means $\mu_{U}, \mu_{V}$ and covariances $\Sigma_{U}$ and $\Sigma_{V}$ as, i.e.,

$U\sim N\left( \mu_{U},\Sigma_{U} \right), V\sim N\left( \mu_{V},\Sigma_{V} \right),$ (S31)

the 2-Wasserstein distance can be explicitly formulated as (See Section J of the Supplementary Information for a detailed proof):

$W_{2}^{2}\left( U,V \right)=\left\| \mu_{U}-\mu_{V} \right\|_{2}^{2}+\mathrm{trace}\left( \sum_{U}+\sum_{V}-2\sqrt{\sqrt{\sum_{U}}\sum_{V}\sqrt{\sum_{U}}} \right).$ (S32)

The optimal transport map is $T\left( x \right)={\mu_{V}+\Sigma}_{U}^{-\frac{1}{2}}\left( \Sigma_{U}^{\frac{1}{2}}\Sigma_{V}\Sigma_{U}^{\frac{1}{2}} \right)^{\frac{1}{2}}\Sigma_{U}^{\frac{1}{2}}(x-\mu_{U})$. Such a Wasserstein distance can capture distributional shifts and model alterations in the structural characteristics of the underlying graph indicated by the covariance matrix. Therefore, our proposed sPGGM is capable of serving as a robust indicator of the critical transition by assessing the dynamic differences between the Gaussian distributions of the normal and pre-disease stages in terms of the Gaussian graphical model.

**A quantitative approach to identify the pre-disease stage based on sPGGM**

Using a set of reference samples/cells obtained from a relatively healthy condition, the proposed sPGGM was employed to identify the pre-disease stage or critical state from a sample-specific/cell-specific perspective. The details of this process are comprehensively outlined in the following sections.

[Step 1] Construction of global gene graph related to the protein-protein interaction (PPI) network. The PPI network is utilized to construct the gene graph, represented as $G=(V,E)$, where $V$ denotes the set of genes after excluding any isolated nodes, and $E$ stands for the interaction of genes with a confidence score of at least 0.9. Additionally, we performed a comparison of the dynamic performance of sPGGM at different confidence thresholds, and found that a threshold of 0.9 yields better results (Figure S15). Moreover, the adjacency matrix $M$ of gene graph $G$ can be derived, where the elements $M$($i, j$) $\in[0, 1]$ indicate whether a regulatory relationship exists between genes $i$ and $j$, with $M$($i, j)=1$ if the edge($i, j$) belongs to $E$.

[Step 2] Extraction of each local graph/network from the global gene graph $G$. Specifically, each local graph can be derived from the global gene graph $G$.For a given gene $g^{k}$, the corresponding $g^{k}$-local graph ${LG}^{k}$ is centered around the gene itself, with its 1st-order neighbors $\left\{ g_{1}^{k}, g_{2}^{k},\ldots, g_{Q}^{k} \right\}$ constituting the $Q$ edges of the local graph. Consequently, if there are $N$ genes in global graph $G$, a total of $N$ distinct local graphs can be obtained. The interactions between the nodes in the $g^{k}$-local graph ${LG}^{k}$ are represented by the adjacency matrix $M^{k}$. A comprehensive explanation for focusing on 1st-order interactions or neighbors and utilizing all local graph/network derived from the global graph $G$ can be found in Section L of the Supplementary Information.

[Step 3] Fitting of a baseline graphical distribution and perturbed graphical distribution for each local graph. Specifically, for a $g^{k}$-local graph ${LG}^{k}$, its multivariate Gaussian graphical distribution $N_{\mathrm{Re}}^{k}(\boldsymbol{\mu}_{\mathbf{Re}}^{\boldsymbol{k}},\sum_{\mathrm{Re}}^{k})$(baseline distribution), with $\boldsymbol{\mu}_{\mathbf{Re}}^{\boldsymbol{k}}$​ as a $d^{k}$-dimensional mean vector and $\sum_{\mathrm{Re}}^{k}$ as a $d^{k}\times d^{k}$ covariance matrix ($d^{k}=Q+1$ indicating the number of genes in the local graph), is determined by the expressions of the $g^{k}$-local graph $\{g^{k}, g_{1}^{k}, g_{2}^{k},\ldots, g_{Q}^{k}\}$ based on the reference samples. Similarly, the perturbed graphical distribution $N_{\mathrm{Per}}^{k}(\boldsymbol{\mu}_{\mathbf{Per}}^{\boldsymbol{k}},\sum_{\mathrm{Per}}^{k})$ for the $g^{k}$-local graph ${LG}^{k}$ can be fitted from the expressions of $\{g^{k}, g_{1}^{k}, g_{2}^{k},\ldots, g_{Q}^{k}\}$ based on mixed samples (consisting of a specific case sample $S_{t}$ and reference group). Moreover, to offer a more accurate representation of the true graphical distribution by incorporating both data information and actual biological molecular associations, the covariance matrix $\tilde{\sum}_{\mathrm{Re}}^{k}$ of baseline graphical distribution and the covariance matrix $\tilde{\sum}_{\mathrm{Per}}^{k}$ of the perturbed graphical distribution are defined by:

$\tilde{\sum}_{\mathrm{Re}}^{k}$= $\sum_{\mathrm{Re}}^{k}⨀M^{k}$, $\tilde{\sum}_{\mathrm{Per}}^{k}$= $\sum_{\mathrm{Per}}^{k}⨀M^{k}$, (S33)

where the symbol $⨀$ indicates a the Hadamard product, and $M^{k}$ represents the adjacency matrix corresponding to the $g^{k}$-local graph ${LG}^{k}$. In addition, it was found that if the degree of the local graph increases, the difference between its baseline and perturbed distributions decreases at the critical point (Figure S16).

[Step 4] Computation of a sample-specific sPGGM score for the case sample $S_{t}$ at a time point $t$. First, the local sPGGM score for each local graph can be calculated as follows. For the $g^{k}$-local graph ${LG}^{k}$, the local sPGGM ${LD}^{k}$, as defined by the following Eq.(S34), is calculated by measuring the 2-Wasserstein (as described in Eq.(S32)) between its baseline graphical distribution $N_{\mathrm{Re}}^{k}(\boldsymbol{\mu}_{\mathbf{Re}}^{\boldsymbol{k}},\tilde{\sum}_{\mathrm{Re}}^{k})$ and perturbed graphical distribution $N_{\mathrm{Per}}^{k}(\boldsymbol{\mu}_{\mathbf{Per}}^{\boldsymbol{k}},\tilde{\sum}_{\mathrm{Per}}^{k})$,

${LD}^{k}=\frac{1}{d^{k}}\left\| \boldsymbol{\mu}_{\mathbf{Re}}^{\boldsymbol{k}}-\boldsymbol{\mu}_{\mathbf{Per}}^{\boldsymbol{k}} \right\|_{2}^{2}+\frac{1}{d^{k}}\mathrm{trace}\left( \tilde{\sum}_{\mathrm{Re}}^{k}+\tilde{\sum}_{\mathrm{Per}}^{k}-2\sqrt{\sqrt{\tilde{\sum}_{\mathrm{Re}}^{k}}\tilde{\sum}_{\mathrm{Per}}^{k}\sqrt{\tilde{\sum}_{\mathrm{Re}}^{k}}} \right).$ (S34)

Then, based on a group of genes exhibiting the highest local sPGGM scores, the sample-specific sPGGM score $D_{t}$ for the particular sample $S_{t}$ is computed using the following formula.

$D_{t}=\frac{1}{L}\sum_{k=1}^{L} {LD}^{k},$ (S35)

where $L$ denotes a configurable parameter set to the count of the top 5% of genes with the highest local sPGGM score. On basis of DNB theory, DNB molecules demonstrate notable collective behaviors, marked by significant fluctuations and strong correlations, as a complex system approaches a pre-disease stage. Therefore, the graphical distributions of local networks with DNB molecules in the critical state show differ markedly from those in the normal stage, potentially causing a sudden increase in the sPGGM score.

[Step 5] Determination of the pre-disease stage through the one-sample test. In this study, we utilized the one-sample test to assess whether the sPGGM score can capture statistically significantly critical dynamics between the normal and pre-disease stages [24]. More details are available in Section M of the Supplementary Information.

**Data preparation and functional study**

The sPGGM presented in this paper has been utilized to a numerical simulation and nine real-world datasets, including an influenza data (GEO: GSE30550), two single-cell data (GABAergic interneurons dataset [GEO: GSE93593] and CD8+ T-cell exhaustion dataset [GSE108989]), and six cancer datasets from the TCGA database: COAD, THCA, KIRC, UCEC, KIRP, and LIHC. For the TCGA datasets, each tumour data includes both tumour and tumour-adjacent samples. Tumour samples were categorized by stage information, with those lacking complete stage data excluded, while adjacent non-tumour samples, reflecting a relatively healthy state, served as the reference group for analysis. For single-cell data, the cells from the first time point can be treated as the reference group, representing early-stage conditions that are typically regarded as a more stable, healthy state before any observable transitions or perturbations occur. More comprehensive details about the sample collection are provided in Section NO of the Supplementary Information. In single-cell datasets, Seurat pipelines were used to process the downstream analysis. For all datasets, a filtering step is conducted to remove probes without corresponding NCBI Entrez gene symbols, as well as genes with excessively low expression levels. Datasets with GSE numbers are available on the NCBI GEO database (https://www.ncbi.nlm.nih.gov/geo), while TCGA datasets can be found through the National Cancer Institute's portal (https://portal.gdc.cancer.gov).

KEGG pathway analysis was executed based on the Kyoto Encyclopedia of Genes and Genomes (https://www.kegg.jp) platform. Functional enrichment analysis was carried out using tools from the Gene Ontology Consortium (http://geneontology.org), ClusterProfiler package [25] and Metascape [26]. Gene function annotations were sourced from GeneCards (http://www.genecards.org/). Protein–Protein Interaction (PPI) networks were accessed through STRING (https://string-db.org/) and visualized with Cytoscape (https://cytoscape.org/).

**O.** **Description of the** **nine real biological datasets**

To demonstrate the capabilities of the sPGGM method, it has been utilized on nine real biological datasets: COAD (Colon Adenocarcinoma), THCA (Thyroid Carcinoma), KIRC (Kidney Renal Clear Cell Carcinoma), UCEC (Uterine Corpus Endometrial Carcinoma), KIRP (Kidney Renal Papillary Cell Carcinoma), and LIHC (Liver Hepatocellular Carcinoma) obtained from The Cancer Genome Atlas (TCGA) database (https://portal.gdc.cancer.gov), as well as influenza infection data (accession number: GSE30550) and single-cell data related to GABAergic interneurons (accession number: GSE93593) and T-cell exhaustion in colorectal cancer from the Gene Expression Omnibus (accession number: GSE108989) sourced from the Gene Expression Omnibus database (http://www.ncbi.nlm.nih.gov/geo). The datasets are described in detail below, along with their sources.

The TCGA-COAD dataset includes 419 tumor samples alongside 41 adjacent tumor samples. Using the clinical data from TCGA, the tumor samples are classified into four stages: stage I (81 samples), stage IIA (145 samples), stage IIB (12 samples), stage IIIA (8 samples), stage IIIB (60 samples), stage IIIC (46 samples), and stage IV (67 samples). Gene expression profiling data can be accessed at: https://portal.gdc.cancer.gov/projects/TCGA-COAD.

The TCGA-THCA dataset contains 511 tumor samples and 59 adjacent tumor samples. According to the clinical information provided by TCGA, these tumor samples can be divided into several stages: stage I (289 samples), stage II (52 samples), stage III (113 samples), and stage IV (57 samples). The gene expression profiling data can be download at: https://portal.gdc.cancer.gov/projects/TCGA-THCA.

The TCGA-KIRC dataset comprises 539 tumor samples and 72 samples from tumor-adjacent tissues. Utilizing the relevant clinical data from TCGA, the tumor samples are categorized into four stages: stage I (274 samples), stage II (59 samples), stage III (123 samples), and stage IV (83 samples). The gene expression data can be download at: https://portal.gdc.cancer.gov/projects/TCGA-KIRC.

The TCGA-UCEC dataset consists of 434 tumor samples and 35 adjacent tumor samples. Based on the corresponding clinical information of TCGA, tumor samples are classified into different stages: stage IA (167 samples), stage IB (148 samples), stage IC (25 samples), stage IIA (6 samples), stage IIB (13 samples), stage IIIA (40 samples), stage IIIB (6 samples), and stage IV (29 samples). The gene expression profiling data can be accessed at: [https://portal.gdc.cancer.gov/projects/TCGA- UCEC](https://portal.gdc.cancer.gov/projects/TCGA-LUAD) .

The TCGA-KIRP dataset comprises 273 tumor samples and 32 adjacent tumor samples. According to the clinical data available from TCGA, the tumor samples are divided into different stages: stage I (186 samples), stage II (29 samples), stage III (44 samples), and stage IV (14 samples). The gene expression profiling data can be obtained from: https://portal.gdc.cancer.gov/projects/TCGA-KIRP.

The TCGA-LIHC dataset contains 349 tumor samples and 50 tumor-adjacent samples. Based on the available clinical data from TCGA, the tumor samples are organized into four stages: stage I (172 samples), stage II (87 samples), stage III (85 samples), and stage IV (5 samples). The gene expression profiling data is available for download at: https://portal.gdc.cancer.gov/projects/TCGA-LIHC.

The influenza infection dataset [27] consists of gene expression profiles obtained from peripheral blood samples of 17 volunteers infected with the H3N2/Wisconsin virus via intranasal administration. Out of these participants, nine exhibited severe infection symptoms (subjects 1, 5, 6, 7, 8, 10, 12, 13, and 15), while the other eight remained healthy. Gene expression measurements were taken at 16 different time points (-24, 0, 5, 12, 21, 29, 36, 45, 53, 60, 69, 77, 84, 93, 101, and 108 hours). For each individual, the gene expression data from the initial four time points (-24, 0, 5, and 12 h) were designated as reference samples, while the data from the subsequent time points were treated as case samples. The gene expression profiling data is available in the Gene Expression Omnibus (GEO) database under the accession number GSE30550.

The GABAergic interneurons dataset [28] describes a protocol for producing cortical interneurons from H1 human embryonic stem cells (hESCs). It includes single-cell RNA sequencing (scRNA-seq) data from hESC-derived, MGE-like cells collected at various time points: D24 (476 cells), D54 (585 cells), D100 (519 cells), and D125 (153 cells) over a 125-day differentiation process that generated multiple ventrally-derived cell types. The gene expression profiling data can be accessed in the Gene Expression Omnibus (GEO) database under accession number GSE93593.

The CD8+ T-cell exhaustion dataset [29] describes the developmental trajectories of CD8+ T-cell exhaustion in colorectal cancer (CRC). The progression of CD8+ T-cell exhaustion is classified into various clusters/stages: stage 1 (358 cells), stage 2 (192 cells), stage 3 (186 cells), stage 4 (191 cells), stage 5 (223 cells), stage 6 (160 cells), and stage 7 (168 cells). The gene expression profiling data can be accessible through the Gene Expression Omnibus (GEO) database under accession number GSE108989.

**References**

[1] Ronen M, Rosenberg R, Alon U *et al*. Assigning numbers to the arrows: parameterizing a gene regulation network by using accurate ex-pression kinetics. *Proc Natl Acad Sci USA* 2002; **99**: 10555–60.

[2] Khanin R, Vinciotti V, Mersinias V *et al*. Statistical reconstruction of transcription factor activity using Michaelis–Menten kinetics. *Biometrics* 2007; **63**: 816–23.

[3] Shi J, Aihara K, Chen L. Dynamics-based data science in biology. *National Science Review* 2021; **8**: nwab029.

[4] Liu R, Chen P, Chen L. Single-sample landscape entropy reveals the imminent phase transition during disease progression. *Bioinformatics* 2020; **36**: 1522-1532.

[5] Liu X, Chang X, Leng S *et al*. Detection for disease tipping points by landscape dynamic network biomarkers. *Natl Sci Rev* 2019; **6**: 775-785.

[6] Liu R, Chen P, Chen L. Single-sample landscape entropy reveals the imminent phase transition during disease progression. *Bioinformatics* 2020; **36**: 1522-1532.

[7] Ren J, Li P, Yan J. CPMI: comprehensive neighborhood-based perturbed mutual information for identifying critical states of complex biological processes. *BMC Bioinformatics* 2024; **25**: 215.

[8] Lyu C, Chen L, Liu X. Detecting tipping points of complex diseases by network information entropy. *Brief Bioinform* 2024; **25**: bbae311.

[9] Reich D, Price AL, Patterson N. Principal component analysis of genetic data. *Nat Genet* 2008; **40**: 491-2.

[10] Zhou P, Wang S, Li T *et al*. Dissecting transition cells from single-cell transcriptome data through multiscale stochastic dynamics. *Nat Commun* 2021; **12**: 5609.

[11] Sha Y, Wang S, Zhou P *et al*. Inference and multiscale model of epithelial-to-mesenchymal transition via single-cell transcriptomic data. *Nucleic Acids Res* 2020; **48**: 9505-9520.

[12] Yang XH, Goldstein A, Sun Y *et al*. Detecting critical transition signals from single-cell transcriptomes to infer lineage-determining transcription factors. *Nucleic Acids Res* 2022; **50**: e91.

[13] Ichiyama K, Long J, Kobayashi Y *et al*. Transcription factor Ikzf1 associates with Foxp3 to repress gene expression in Treg cells and limit autoimmunity and anti-tumor immunity. *Immunity* 2024; **57**: 2043-2060.

[14] Cao J, Spielmann M, Qiu X *et al*. The single-cell transcriptional landscape of mammalian organogenesis. *Nature* 2019; **566**: 496-502.

[15] Tabula Muris Consortium. A single-cell transcriptomic atlas characterizes ageing tissues in the mouse. *Nature* 2020; **583**: 590-595.

[16] Kolouri S, Park S R, Thorpe M *et al*. Optimal mass transport: Signal processing and machine-learning applications. *IEEE signal processing magazine* 2017, **34**: 43-59.

[17] Givens C R and Shortt R M. A class of Wasserstein metrics for probability distributions. *Michigan Mathematical Journal* 1984; **31**: 231-240.

[18] Panaretos V M and Zemel Y. Statistical aspects of Wasserstein distances. *Annual review of statistics and its application* 2019; **6**: 405-431.

[19] Liu R, Li M, Liu ZP *et al*. Identifying critical transitions and their leading biomolecular networks in complex diseases. *Sci Rep* 2012; **2**: 813.

[20] Scheffer M, Carpenter S, Foley JA *et al*. Catastrophic shifts in ecosystems. *Nature* 2001; **413**: 591-6.

[21] Uhler C. Gaussian graphical models: An algebraic and geometric perspective. arXiv: 1707.04345.

[22] Bishop C M and Nasrabadi N M. Pattern recognition and machine learning. New York: springer, 2006.

[23] Zhao H and Duan ZH. Cancer genetic network inference using Gaussian Graphical Models. *Bioinform Biol Insights* 2019; **13**: 1177932219839402.

[24] Yadav S K, Singh S, Gupta R *et al*. Test of inference: One-sample or two-sample mean. Biomedical Statistics: A Beginner's Guide, 2019, 99-105.

[25] Yu G, Wang LG, Han Y *et al*. clusterProfiler: an R package for comparing biological themes among gene clusters. *OMICS* 2012; **16**: 284-7.

[26] Zhou Y, Zhou B, Pache L *et al*. Metascape provides a biologist-oriented resource for the analysis of systems-level datasets. *Nat Commun* 2019; **10**: 1523.

[27] Huang YS, Zaas AK, Rao A *et al*. Temporal dynamics of host molecular responses differentiate symptomatic and asymptomatic influenza a infection. *PloS Genet* 2011; **7**: e1002234.

[28] Close, J. L., Yao, Z., Levi, B. P. *et al*. Single-cell profiling of an in vitro model of human interneuron development reveals temporal dynamics of cell type production and maturation. *Neuron* 2017; **93**: 1035-1048.

[29] Jiaqi Hu, Chongyin Han, Jiayuan Zhong *et al*. Dynamic network biomarker of pre-exhausted CD8+ T cells contributed to T cell exhaustion in colorectal cancer. *Frontiers in Immunology* 2021, **12**: 691142.
